# Exclusive Breastfeeding and Cognition, Executive Function, and Behavioural Disorders in Primary School-Aged Children in Rural South Africa: A Cohort Analysis

**DOI:** 10.1371/journal.pmed.1002044

**Published:** 2016-06-21

**Authors:** Tamsen J. Rochat, Brian Houle, Alan Stein, Hoosen Coovadia, Anna Coutsoudis, Chris Desmond, Marie-Louise Newell, Ruth M. Bland

**Affiliations:** 1 Africa Centre for Population Health, University of KwaZulu-Natal, Mtubatuba, South Africa; 2 Human and Social Development Research Programme, Human Sciences Research Council, Durban, South Africa; 3 Section of Child and Adolescent Psychiatry, Department of Psychiatry, Oxford University, Oxford, United Kingdom; 4 Developmental Pathways to Health Research Unit, School of Clinical Medicine, University of Witwatersrand, Johannesburg, South Africa; 5 MRC/Wits Rural Public Health Transitions Research Unit (Agincourt), School of Public Health, Faculty of Health Sciences, University of the Witwatersrand, Johannesburg, South Africa; 6 School of Demography, The Australian National University, Canberra, Australia; 7 CU Population Center, Institute of Behavioural Science, University of Colorado at Boulder, Boulder, Colorado, United States of America; 8 School of Public Health, Faculty of Health Sciences, University of Witwatersrand, Johannesburg, South Africa; 9 MatCH Health Systems (Maternal, Adolescent and Child Health), KwaZulu-Natal, South Africa; 10 Department of Paediatrics and Child Health, University of KwaZulu-Natal, Durban, South Africa; 11 Human Development and Health, Faculty of Medicine, University of Southampton, Southampton, United Kingdom; 12 Royal Hospital for Sick Children and Institute of Health and Wellbeing, College of Medical, Veterinary and Life Sciences, University of Glasgow, Glasgow, United Kingdom; Makerere University Medical School, UGANDA

## Abstract

**Background:**

Exclusive breastfeeding (EBF) is associated with early child health; its longer-term benefits for child development remain inconclusive. We examine the associations between EBF, HIV exposure, and other maternal/child factors and the cognitive and emotional-behavioural development of children aged 7–11 y.

**Methods and Findings:**

The Vertical Transmission Study (VTS) supported EBF in HIV-positive and HIV-negative women; between 2012 and 2014, HIV-negative VTS children (332 HIV exposed, 574 HIV unexposed) were assessed in terms of cognition (Kaufman Assessment Battery for Children Second Edition [KABC-II]), executive function (Developmental Neuropsychological Assessment Second Edition [NEPSY-II]), and emotional-behavioural functioning (parent-reported Child Behaviour Checklist, [CBCL]). We developed population means by combining the VTS sample with 629 same-aged HIV-negative children from the local demographic platform. For each outcome, we split the VTS sample into scores above or at/below each population mean and modelled each outcome using logistic regression analyses, overall and stratified by child sex. There was no demonstrated effect of EBF on overall cognitive functioning. EBF was associated with fewer conduct disorders overall (adjusted odds ratio [aOR] 0.44 [95% CI 0.3–0.7], *p* ≤ 0.01), and there was weak evidence of better cognition in boys who had been exclusively breastfed for 2–5 mo versus ≤1 mo (Learning subscale aOR 2.07 [95% CI 1.0–4.3], *p* = 0.05). Other factors associated with better child cognition were higher maternal cognitive ability (aOR 1.43 [95% CI 1.1–1.9], *p* = 0.02, Sequential; aOR 1.74 [95% CI 1.3–2.4], *p* < 0.001, Planning subscales) and crèche attendance (aOR 1.96 [95% CI 1.1–3.5], *p* = 0.02, Sequential subscale). Factors positively associated with executive function were home stimulation (aOR 1.36 [95% CI 1.0–1.8], *p* = 0.04, Auditory Attention; aOR 1.35 [95% CI 1.0–1.8], *p* = 0.05, Response Set) and crèche (aOR 1.74 [95% CI 1.0–3.0], *p* = 0.05, Animal Sorting). Maternal mental health problems and parenting stress were associated with increased emotional-behavioural problems on the total CBCL (aOR 2.44 [95% CI 1.3–4.6], *p* = 0.01; aOR 7.04 [95% CI 4.2–11.9], *p* < 0.001, respectively). Maternal HIV status was not associated with any outcomes in the overall cohort. Limitations include the nonrandomised study design and lack of maternal mental health assessment at the child’s birth.

**Conclusions:**

EBF was associated with fewer than average conduct disorders and weakly associated with improved cognitive development in boys. Efforts to improve stimulation at home, reduce maternal stress, and enable crèche attendance are likely to improve executive function and emotional-behavioural development of children.

## Abstract 2

Bland and colleagues report that exclusive breastfeeding leads to a reduction in conduct disorder incidents in children but does not significantly impact on cognition.

## Author Summary

**Why Was This Study Done?:**

- The benefits of exclusive breastfeeding (EBF) in early life are well established and include optimal nutrition and protection from infectious diseases.
- The longer-term benefits of EBF on child development and behaviour are less clear, and studies in low-income settings have shown conflicting results depending on the design of the study and whether other factors known to influence development, such as HIV exposure, have been taken into account.
- There is a dearth of evidence examining the development of HIV-uninfected children born to HIV-infected mothers and whether these children are disadvantaged compared to those born to HIV-uninfected mothers.

**What Did the Researchers Do and Find?:**

- This study was established in 2012 to investigate the development (cognitive and emotion-behaviour) of 1,536 HIV-negative children, born to HIV-infected and HIV-uninfected mothers, in rural South Africa, taking into account a range of current and early life factors known to be associated with child development.
- Duration of EBF was associated with a reduction in conduct disorders in girls and boys, but there was no association with cognitive development in the overall sample, when allowing for other factors. Maternal intelligence quotient (IQ) was strongly associated with children’s later cognitive development.
- Children born to HIV-infected mothers performed as well as the children born to HIV-uninfected mothers.

**What Do the Findings Mean?:**

- Promoting, protecting, and supporting EBF may result in fewer conduct disorders, in addition to the established benefits of improved nutrition and reduced morbidity and mortality.
- Reducing conduct disorders is important because they may lead to aggressive or disruptive behaviours and are associated with an increase in later criminal behaviour and poor long-term mental health and academic achievement.

## Introduction

There is strong evidence that exclusive breastfeeding (EBF) for 6 mo, as recommended by the World Health Organization (WHO) [1], optimises infant nutrition and substantially decreases mortality and morbidity from infectious diseases [2,3]. The relationship between EBF and cognitive development is less clear [4,5], although studies in high-income settings [6–8], a randomised trial from Belarus [9], and a recent study from Brazil [10] have shown positive associations. A large systematic review showed conflicting results depending on the study design and rigour, as well as the number of factors adjusted for [4]. The few studies from resource-limited settings were almost twice as likely to find no association. This suggests that confounding variables, including socioeconomic status and maternal cognitive ability, affect the choice to breastfeed and the positive effects found. Measuring the duration of EBF accurately is challenging because of factors related to definition, timing, and duration of recall [11,12], and many of the studies were limited by poor documentation of breastfeeding patterns [13,14]. Further limitations included small sample sizes [15,16] and predominantly Caucasian populations, with only one small study from Africa [16], which found no association with cognitive development but some advantages for child behaviour in breastfed infants. There was no evidence from HIV-prevalent areas where the long-term effect of EBF on child development remains unquantified.

Studies exploring the link between EBF and later development have focused on core cognitive development, sometimes termed the intelligence quotient (IQ). However, higher-order cognitive function, termed executive function, is critical for later development, particularly the ability to function in society [17]. Executive function coordinates and controls information processing, which is important for a child’s ability to manage emotions and behaviour, to follow rules, to concentrate, and to form friendships. Thus, executive function influences educational and social success [18]. Executive function is susceptible to environmental influences and therefore an important intervention target [19]. In addition, few breastfeeding studies have examined emotional-behavioural development, an important outcome affected by early life factors, which predicts later educational achievement.

The Vertical Transmission Study (VTS) (2001–2006) supported HIV-positive and HIV-negative women to practice EBF in a rural area of South Africa before antiretroviral treatment became available [20], providing the first evidence that EBF reduced the risk of postnatal HIV transmission [21] and was associated with significant benefits for children’s health and growth until up to 2 y of age [22,23] (Registration: NCT01948557, National Institute of Health, ClinicalTrials.gov). Here we investigate the association between EBF, HIV exposure, and other early and current life factors and later cognitive development, executive function, and emotional-behavioural development in VTS children now aged 7 to 11 y. We accounted for maternal cognitive function, home stimulation, crèche attendance, and maternal/caregiver stress and mental health and hypothesized that EBF would result in improved longer-term development in children, despite exposure to HIV and poverty.

## Methods

Ethics permission for this study was granted by the Biomedical Research Ethics Committee (BREC), University of KwaZulu-Natal, South Africa (BF184/12). Women were contacted by telephone or a home visit to ask if they would be interested in this study. Those who agreed were then visited by a field worker who explained, and provided written details of, the study and obtained written informed consent from the mother and primary caregiver (if this was not the child’s mother).

The VTS, a nonrandomised, prospective, intervention cohort study, was implemented between 2001 and 2006 from the Africa Centre for Population Health, which also hosts a Demographic Surveillance System (DSS) platform [24]. This lay-counsellor, home-based intervention was designed to support mothers to practice EBF for the first 180 d of life [25]. Between 2012 and 2014, we re-enrolled HIV-negative children (aged 7–11 y) born to HIV-positive (“exposed”) and HIV-negative (“unexposed”) mothers from the VTS cohort; HIV-infected children were not re-enrolled because they have different developmental trajectories [26].

To establish a comparative population mean for the developmental outcomes (in the absence of appropriate normative data for validated cognitive assessments), we assessed 630 (485 unexposed; 145 exposed) same-aged HIV-negative children from the DSS, not included in the VTS, and combined these with the VTS sample. Mothers in the DSS group were exposed to the same antenatal care at local clinics, including messages regarding HIV and infant feeding, but not to the home-based intervention to support EBF. We aimed to rerecruit all available 1,289 VTS children meeting the inclusion criteria, of whom 935 (75%) were enrolled and 906 (70%) fully assessed. To establish a robust population mean, we used the population platform of the Africa Centre for Population Health surveillance to identify all 1,226 resident children who were matched for age and HIV exposure to the VTS children but had not been exposed to the VTS intervention. Of these, 844 children met eligibility criteria, 657 (77%) enrolled, and 630 (75%) completed assessments.

This analysis includes the developmental outcomes of the VTS children, for all of whom we have accurate data on infant feeding and HIV exposure; their outcomes are related to the population means. Children were enrolled if both the mother and child were alive, the child was a resident in the research area, the mother and child’s current HIV status was known, and, for the DSS children, if the HIV status during pregnancy was known, the mother received antenatal services in the study community, and the maternal-held child Road-to-Health Card was available.

### Outcome Measures

#### Child cognition

To assess the cognitive development, we used the full Kaufman Assessment Battery for Children (KABC-II) [27]. This has four subscales, each with subtests, which measure audio and visual memory and memory span (“Sequential Processing”); spatial and visual perception, reasoning, and maths ability (“Simultaneous Processing”); focused and selective attention and the ability to store auditory and visual stimuli simultaneously (“Learning Ability”); and decision-making ability (“Planning”). There is an additional subtest to assess reasoning and language development (“Riddles”). After discussion with experts, including the authors of the KABC-II, to be more culturally appropriate, we substituted a subtest in the Learning Ability subscale (“Atlantis”/ “Atlantis delayed” replaced “Rebus”/ “Rebus delayed”). The KABC-II has been used in low-middle-income countries and validated in Africa [28] (Cronbach’s α = 0.75).

#### Child executive function

Three subtests from the executive function domain of NEPSY-II [29] were used: Animal Sorting (inhibition, planning, and cognitive flexibility), Auditory Attention (vigilance and selective/ sustained auditory attention), and Response Set (inhibition of previously learned stimuli).

All subtest raw scores for both NEPSY-II and KABC-II were transformed to scaled scores, according to the child’s age, using standardised tables published by the test developers.

#### Child emotional and behavioural functioning

We used the parent-reported Child Behaviour Checklist (CBCL) [30], which has been validated across multiple cultural settings [31]. This includes 120-items in two subscales—“Internalising disorders” and “Externalising disorders”—and a composite Total score. A high score indicates more problems. Scores were normed using multicultural Rating-to-Score norming software to produce normed t-tests for the Total score, the two subscales, and the six Diagnostic and Statistical Manual (DSM) disorders: affective, anxious, somatic, attention deficit hyperactivity, oppositional, and conduct disorders (Cronbach’s α = 0.94).

### Maternal Mental Health Measures

All psychometric measures had been previously used in the population; clinical algorithms for depression and anxiety were used.

- Depression and anxiety: measured using the Patient Health Questionnaire Depression (PHQ-9) and Generalized Anxiety Disorder 7-item (GAD-7) scales [32,33], identifying depressive or anxiety symptoms with symptom frequency and severity.
- Alcohol: the WHO Alcohol Use Disorders Identification Test (AUDIT-6) assessed alcohol use and severity [34].
- Parenting stress: measured using the Parenting Stress Index Short Form (PSI-36), a 36-item scale measuring stress related to the parental role, the parent–child relationship, and the degree to which the parent finds the child difficult [35].

The home environment was assessed using a locally adapted version of the Home Observation for Measurement of the Environment (HOME) inventory [36]. Maternal cognitive ability was assessed using the Standard Raven’s Progressive Matrix [37].

### Data Collection

Data were collected over three visits between September 2012 and September 2014. Study consent was obtained at Visit 1, current socioeconomic and health data and mothers’ mental health and cognitive ability at Visit 2, and children’s cognition and executive function at Visit 3. When the mother was not the primary caregiver, mental health assessments were completed by the child’s primary caregiver. Assessments were conducted by graduate-level research assistants with 3–5 y of child developmental assessment experience. The median number of days between Visit 2 and Visit 3 was 18 d.

### Statistical Analyses

Analyses were based on data extracted on 30 October 2014 and conducted using STATA version 13. For each outcome, we calculated a population mean from all VTS and DSS children and then created a binary indicator by splitting the VTS group into those scoring above or at/below the mean. For the HOME assessment and Raven’s score, we created a low/high indicator consisting of equal-size groups by splitting the VTS sample based on their median. In the VTS, daily feeding data were collected at weekly intervals. We defined EBF as the total number of days in the first 6 mo that the child received only breastmilk and then divided this number by 30, into months, irrespective of whether the days were sequential. We previously reported [25] that approximately 40% of VTS women interrupted EBF at some point in the first 6 mo, mostly by giving water or formula milk, of whom approximately 60% returned to EBF within 2 d. We considered that the total number of days of EBF in the first 6 mo was more likely to have an impact on child development than whether the days were sequential or not, and we did not wish to exclude children who had received breastmilk for nearly all 180 d except for 1 or 2 d when they received breastmilk and other fluids. Based on the existing literature and theoretical and conceptual reasoning, we identified relevant factors, including child, maternal, early life, socioeconomic, and household factors related to child development; we did not apply any stepwise regression techniques. For selection of the most relevant socioeconomic variable, we used principal components analysis to identify the top variables that explained the overall variance. We modelled each of the outcomes using complete case logistic regression analyses, accounting for intramother correlation (for twins). We included child sex, child age, birth order, and maternal age; early life factors including birthweight, maternal HIV status during pregnancy, months of EBF, urban/rural residence, ownership of a fridge (wealth indicator), maternal education, and whether the mother was the main income provider at the time of the child’s birth; and other factors including maternal current HIV status, cognitive ability, mental health and parenting stress, crèche exposure, current indicators of perception of household wealth, and HOME assessment score. Sex differences in cognitive development exist at the primary school age [38], and we also estimated sex-stratified logistic regression models, using the same outcomes.

We explored several approaches to modelling the developmental data, including continuous outcomes (see S1 Table) and upper versus lower quintiles, as well as other methods of categorising the EBF data, including cumulative, sequential, days of EBF, and ever/never EBF, but the results were not substantially different.

## Results

### The Sample

**Fig 1:**
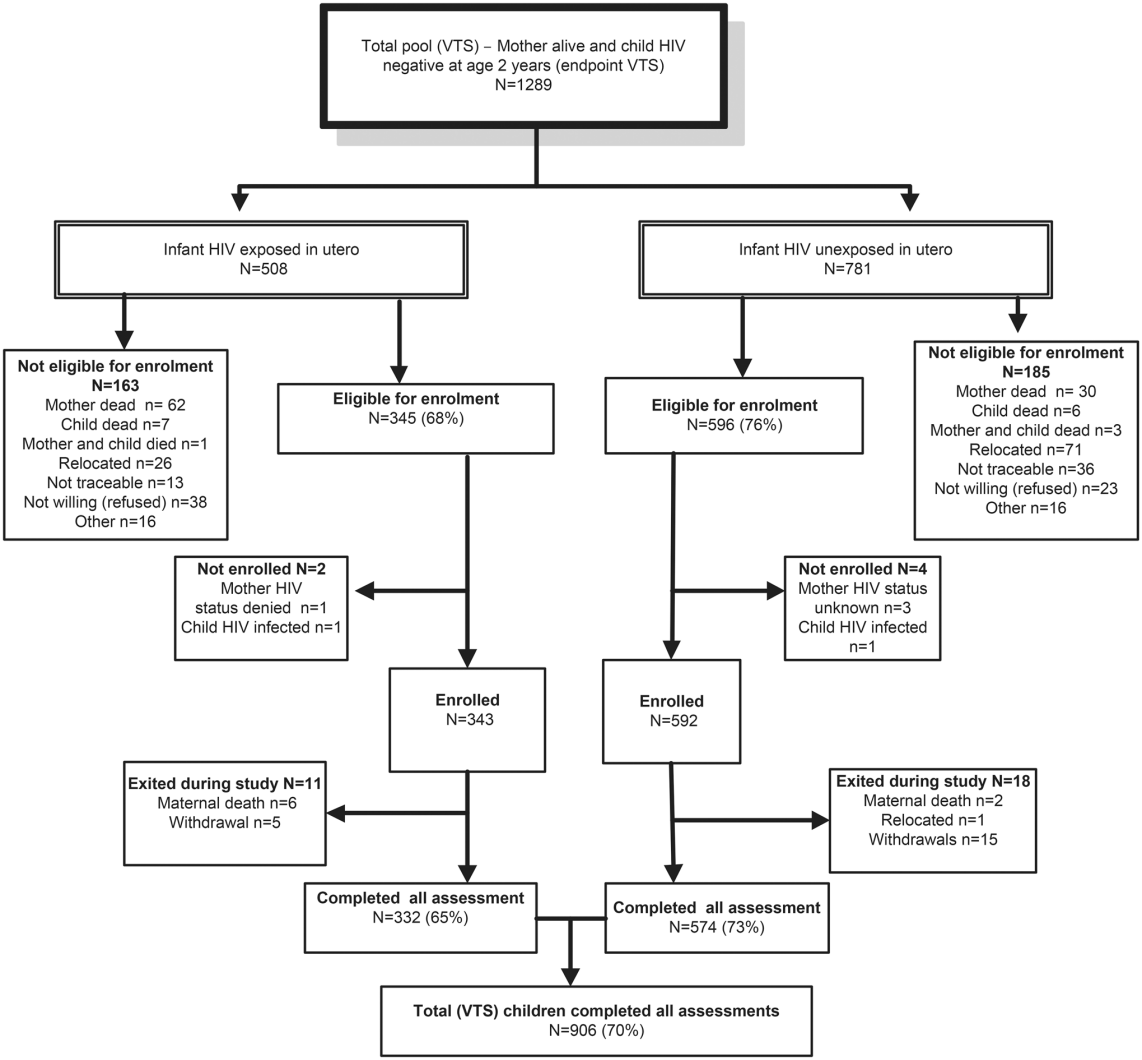
Consort diagram of VTS HIV-uninfected children included in the current follow-up.

By end of the VTS follow-up in September 2006, when children were aged 2 y, 1,289 HIV-negative children were alive, of whom 941 were eligible for re-enrolment and 906 were assessed (Fig 1). Compared to HIV-unexposed children, exposed children were more likely to have a mother who was older and the main income provider, less likely to have been exclusively breastfed until 6 mo of age and to have attended crèche, and more likely to have a primary caregiver with a current mental health disorder (Table 1). Compared to children included in the current analysis, the 383 VTS children excluded were more likely to have a younger HIV-uninfected mother with more years of education at the time of pregnancy and were less likely to have a low birthweight (Table 1).

**Table 1:** Characteristics of children and mothers included and excluded from the analyses of the Vertical Transmission Study (VTS) cohort.

	Total VTS Included (*n* = 906) by HIV Exposure			Total VTS (*n* = 1,289) Included and Excluded		
Variable	*Unexposed*	*Exposed*	*p*-Value	*Included*	*Excluded* ^f^	*p*-Value
	*n* = 574 (63%)	*n* = 332 (37%)		*n* = 906 (70%)	*n* = 383 (30%)	
***Child Sex***						
Female	288 (50.2)	169 (50.9)	0.832	457 (50.4)	198 (51.7)	0.680
Male	286 (49.8)	163 (49.1)		449 (49.6)	185 (48.3)	
***Child Age (Current)***						
8 y	23 (4.0)	20 (6.0)	**<0.001**	43 (4.8)		
9 y	289 (50.3)	114 (34.4)		403 (44.5)		
10 y	238 (41.5)	140 (42.3)		378 (41.8)		
11 y	24 (4.2)	57 (17.2)		81 (9.0)		
Missing	0	1		1		
***Mother’s Age (at Birth*** ^a^ ***)***						
Less than 20 y	154 (26.8)	34 (10.2)	**<0.001**	187 (20.6)	101 (26.4)	**<0.001**
20–29 y	249 (43.4)	190 (57.2)		442 (48.8)	215 (56.1)	
30+ y	171 (29.8)	108 (32.5)		277 (30.6)	67 (17.5)	
***Mother’s Education (at Birth)***						
None	47 (8.2)	21 (6.3)	0.535	68 (7.5)	23 (6.0)	**0.002**
Primary	216 (37.6)	137 (41.3)		353 (39.0)	113 (29.5)	
Some secondary	207 (36.1)	111 (33.4)		318 (35.1)	149 (38.9)	
Completed secondary and postsecondary	104 (18.1)	63 (19.0)		167 (18.4)	98 (25.6)	
***Birthweight***						
Low birthweight^b^	47 (8.5)	42 (13.7)	**0.018**	89 (10.4)	22 (6.1)	**0.017**
Normal birthweight	503 (91.5)	265 (86.3)		768 (89.6)	340 (93.9)	
Missing	24	25		49	21	
***Exclusive Breastfeeding*** ^c^						
0–1 mo	44 (7.7)	67 (20.2)	**<0.001**	111 (12.3)	49 (12.8)	0.460
2–5 mo	167 (29.1)	101 (30.5)		268 (29.6)	125 (32.7)	
6 mo	363 (63.2)	163 (49.2)		526 (58.1)	208 (54.5)	
Missing	0	1		1	1	
***Birth Order***						
Birth order 1–2	346 (60.3)	176 (53.2)	**0.015**	522 (57.7)		
Birth order 3–4	115 (20.0)	94 (28.4)		209 (23.1)		
Birth order 5+	113 (19.7)	61 (18.4)		174 (19.2)		
Missing	0	1		1		
***Mother’s HIV Status***						
Negative	403 (70.5)	0 (0.0)	**<0.001**	403 (44.6)	207 (54.0)	**0.002**
Positive pregnancy	0 (0.0)	332 (100.0)		332 (36.7)	176 (46.0)	
Positive since pregnancy	169 (29.5)	0 (0.0)		169 (18.7)		
Missing	2	0		6	0	
***Residence (at Birth)***						
Rural	377 (65.7)	177 (53.3)	**<0.001**	554 (61.1)	241 (62.9)	0.549
Urban	197 (34.3)	155 (46.7)		352 (38.9)	142 (37.1)	
***Main Income (at Birth)***						
Other	539 (94.1)	286 (86.4)	**<0.001**	825 (91.3)	355 (92.9)	0.319
Mother	34 (5.9)	45 (13.6)		79 (8.7)	27 (7.1)	
Missing	1	1		2	1	
***Owns Fridge (at Birth)***						
Fridge: No	328 (57.2)	204 (61.6)	0.196	532 (58.8)	220 (57.6)	0.676
Fridge: Yes	245 (42.8)	127 (38.4)		372 (41.2)	162 (42.4)	
Missing	1	1		2	1	
***Perception of Wealth (Current)***						
Very comfortable	56 (9.8)	30 (9.1)	0.860	86 (9.5)		
Getting by	340 (59.2)	202 (61.0)		542 (59.9)		
Extremely poor	178 (31.0)	99 (29.9)		277 (30.6)		
Missing	0	1		1		
***Crèche*** ^d^						
No crèche	37 (6.4)	35 (10.5)	**0.028**	72 (7.9)		
Attended crèche	537 (93.6)	297 (89.5)		834 (92.1)		
***Maternal Mental Health*** ^e^ ***(Current)***						
No mental disorders	525 (91.5)	289 (87.3)	**0.045**	814 (89.9)		
Depression or anxiety or alcohol use	49 (8.5)	42 (12.7)		91 (10.1)		
Missing	0	1		1		
***Parenting Stress (Current)***						
Parenting stress ≤90	493 (85.9)	280 (84.3)	0.525	773 (85.3)		
Parenting stress ≥90	81 (14.1)	52 (15.7)		133 (14.7)		

Footnotes apply to Tables 1–9. Bold numbers indicate a *p*-value < 0.05

^a^ “Birth” indicates that the variable relates to when the child was born as opposed to the time of follow-up at 7–11 y; “Current” indicates that the variable relates to the time of follow-up in this study when the child was aged between 7–11 y.

^b^ Low birthweight was defined as <2.5 kg.

^c^ Exclusive breastfeeding (EBF) for this analysis was defined as the number of days when the child only received breastmilk and no other fluids or solids; the total number of days was divided by 30 to categorise the number of months of EBF.

^d^ Crèche is a noncompulsory, nongovernmental preschool; children start primary school in South Africa at the age of 7 y.

^e^ A provisional depression diagnosis determined by the PHQ-9 diagnostic algorithm required at least one Criteria A (mood and loss of interest) and 2–4 Criteria B (weight, sleep, agitation/retardation, fatigue, guilt, concentration, and suicidality) for more than half the days. A provisional anxiety diagnosis required meeting Criteria A (anxiety) and ≥3 Criteria B (worry, restlessness, fatigue, concentration, irritability, and sleep) for more than half the days.

^f^ Child age and birth order are not shown for the excluded children, some of whom died or were lost to follow-up. Other variables omitted for the excluded children are factors measured since the VTS cohort, including current perception of wealth, attendance at crèche, caregiver mental health, and parenting stress.

### Cognitive Outcomes

None of the cognitive development measurements were significantly associated with EBF or maternal HIV status in adjusted analyses (Table 2). In multivariable analyses, the only variable significantly positively associated with performance on all cognitive subscales was maternal cognitive ability (measured using the Standard Raven’s Progressive Matrix) (Table 2). Boys were approximately 30% less likely than girls to score above the mean in the Sequential Processing subscale, which tests audio and visual memory and memory span (adjusted odds ratio [aOR] 0.71 [95% CI 0.5–0.9], *p* = 0.03), whilst children who had attended crèche were almost twice as likely to score above the mean (aOR 1.96 [95% CI 1.1–3.5], *p* = 0.02). Children who were older at assessment performed worse on Riddles (aOR 0.40 [95% CI 0.2–1.0], *p* = 0.05).

**Table 2:** Factors associated with children’s cognitive outcomes measured by the Kauffman Assessment Battery (KABC-II).

	Sequential (*n* = 825)		Planning (*n* = 825)		Learning (*n* = 825)		Simultaneous (*n* = 825)		Riddles (*n* = 824)	
	OR (CI)	AOR (CI)	OR (CI)	AOR (CI)	OR (CI)	AOR (CI)	OR (CI)	AOR (CI)	OR (CI)	AOR (CI)
***Child Sex***										
Female	1.00	1.00	1.00	1.00	1.00	1.00	1.00	1.00	1.00	1.00
Male	**0.69** ** **(0.5–0.9)**	**0.71** * **(0.5–0.9)**	**0.74** * **(0.6–1.0)**	0.77 (0.6–1.0)	1.13 (0.9–1.5)	1.18 (0.9–1.6)	1.22 (0.9–1.7)	1.26 (0.9–1.7)	0.92 (0.7–1.2)	0.91 (0.7–1.2)
***Child Age (Current)***										
8 y	1.00	1.00	1.00	1.00	1.00	1.00	1.00	1.00	1.00	1.00
9 y	1.06 (0.5–2.1)	1.16 (0.6–2.3)	1.12 (0.6–2.0)	1.29 (0.7–2.4)	1.20 (0.6–2.3)	1.17 (0.6–2.3)	0.66 (0.4–1.2)	0.68 (0.4–1.3)	0.84 (0.4–1.6)	0.99 (0.5–2.0)
10 y	0.81 (0.4–1.6)	0.93 (0.5–1.9)	0.96 (0.5–1.8)	1.21 (0.6–2.3)	1.06 (0.6–2.0)	1.02 (0.5–2.0)	**0.47** * **(0.3–0.9)**	**0.49** * **(0.3–1.0)**	0.59 (0.3–1.1)	0.70 (0.3–1.4)
11 y	0.75 (0.3–1.7)	0.91 (0.4–2.1)	0.60 (0.3–1.3)	0.85 (0.4–1.9)	1.12 (0.5–2.4)	1.17 (0.5–2.6)	0.53 (0.2–1.1)	0.57 (0.3–1.3)	**0.34** * **(0.1–0.8)**	**0.40** * **(0.2–1.0)**
***Mother’s Age (at Birth)***										
Less than 20 y	1.00	1.00	1.00	1.00	1.00	1.00	1.00	1.00	1.00	1.00
20–29 y	1.03 (0.7–1.5)	1.01 (0.7–1.5)	0.88 (0.6–1.3)	0.81 (0.5–1.2)	1.14 (0.8–1.7)	1.16 (0.8–1.8)	1.26 (0.9–1.8)	1.11 (0.7–1.7)	1.24 (0.8–1.8)	1.08 (0.7–1.7)
30+ y	1.11 (0.7–1.7)	1.52 (0.8–2.7)	0.82 (0.6–1.2)	0.80 (0.4–1.4)	1.21 (0.8–1.8)	1.90 (1.0–3.4)	1.37 (0.9–2.0)	1.16 (0.6–2.1)	1.12 (0.7–1.7)	1.09 (0.6–2.0)
***Maternal IQ (Current)*** ^a^										
Low Raven’s	1.00	1.00	1.00	1.00	1.00	1.00	1.00	1.00	1.00	1.00
High Raven’s	**1.60** ** **(1.2–2.1)**	**1.43** * **(1.1–1.9)**	**2.04** *** **(1.5–2.7)**	**1.74** *** **(1.3–2.4)**	**1.84** *** **(1.4–2.4)**	**1.64** ** **(1.2–2.2)**	**1.60** ** **(1.2–2.1)**	**1.46** * **(1.0–2.0)**	**1.81** *** **(1.4–2.4)**	**1.60** ** **(1.2–2.2)**
***Mother’s Education (at Birth)***										
None	1.00	1.00	1.00	1.00	1.00	1.00	1.00	1.00	1.00	1.00
Primary	0.96 (0.5–1.7)	0.97 (0.5–1.8)	1.00 (0.6–1.8)	1.05 (0.6–1.9)	1.11 (0.6–2.0)	0.97 (0.5–1.8)	1.31 (0.7–2.3)	1.31 (0.7–2.4)	0.93 (0.5–1.7)	0.92 (0.5–1.8)
Some secondary	1.23 (0.7–2.2)	1.13 (0.6–2.2)	1.50 (0.8–2.7)	1.42 (0.8–2.7)	1.34 (0.7–2.4)	1.12 (0.6–2.1)	1.36 (0.8–2.4)	1.42 (0.7–2.7)	1.38 (0.7–2.6)	1.30 (0.7–2.6)
Completed secondary/postsecondary	1.70 (0.9–3.1)	1.40 (0.7–2.8)	**2.01** * **(1.0–3.7)**	1.66 (0.8–3.4)	**2.21** * **(1.2–4.1)**	1.52 (0.8–3.0)	**2.46** ** **(1.3–4.6)**	**2.15** * **(1.0–4.4)**	**1.99** * **(1.0–3.8)**	1.59 (0.8–3.3)
***Birthweight***										
Low Birthweight	1.00	1.00	1.00	1.00	1.00	1.00	1.00	1.00	1.00	1.00
Normal Birthweight	1.36 (0.9–2.2)	1.34 (0.8–2.2)	1.39 (0.9–2.2)	1.42 (0.9–2.3)	1.42 (0.9–2.2)	1.35 (0.8–2.2)	1.48 (0.9–2.3)	1.48 (0.9–2.4)	1.61 (1.0–2.7)	1.58 (0.9–2.8)
***Exclusive Breastfeeding***										
0–1 mo	1.00	1.00	1.00	1.00	1.00	1.00	1.00	1.00	1.00	1.00
2–5 mo	1.09 (0.7–1.7)	1.27 (0.8–2.1)	0.93 (0.6–1.5)	0.96 (0.6–1.6)	0.93 (0.6–1.5)	1.16 (0.7–1.9)	1.02 (0.6–1.6)	1.34 (0.8–2.2)	0.76 (0.5–1.2)	1.07 (0.6–1.8)
6 mo	1.01 (0.7–1.6)	1.23 (0.8–2.0)	0.75 (0.5–1.1)	0.80 (0.5–1.3)	1.04 (0.7–1.6)	1.29 (0.8–2.1)	0.94 (0.6–1.4)	1.29 (0.8–2.1)	0.77 (0.5–1.2)	1.18 (0.7–1.9)
***Birth Order (Birth)***										
Birth order 1–2	1.00	1.00	1.00	1.00	1.00	1.00	1.00	1.00	1.00	1.00
Birth order 3–4	0.92 (0.7–1.3)	0.87 (0.6–1.3)	0.86 (0.6–1.2)	1.15 (0.8–1.7)	0.91 (0.7–1.3)	0.84 (0.6–1.3)	1.06 (0.8–1.5)	1.08 (0.7–1.6)	1.15 (0.8–1.6)	1.27 (0.8–2.0)
Birth order 5+	0.84 (0.6–1.2)	0.72 (0.4–1.3)	0.87 (0.6–1.2)	1.33 (0.8–2.3)	0.76 (0.5–1.1)	0.61 (0.3–1.0)	1.17 (0.8–1.7)	1.25 (0.7–2.2)	0.81 (0.6–1.2)	1.07 (0.6–2.0)
***Mother’s HIV Status***										
Negative	1.00	1.00	1.00	1.00	1.00	1.00	1.00	1.00	1.00	1.00
Positive pregnancy	1.03 (0.8–1.4)	1.13 (0.8–1.6)	0.75 (0.5–1.0)	0.83 (0.6–1.2)	1.11 (0.8–1.5)	1.23 (0.9–1.7)	0.92 (0.7–1.2)	0.99 (0.7–1.4)	1.10 (0.8–1.5)	1.29 (0.9–1.9)
Positive since pregnancy	0.81 (0.6–1.2)	0.83 (0.5–1.2)	**0.66** * **(0.5–1.0)**	0.69 (0.5–1.0)	1.04 (0.7–1.5)	1.18 (0.8–1.7)	**0.60** * **(0.4–0.9)**	0.69 (0.5–1.0)	0.84 (0.6–1.3)	0.96 (0.6–1.5)
***Residence (at Birth)***										
Rural	1.00	1.00	1.00	1.00	1.00	1.00	1.00	1.00	1.00	1.00
Urban	**1.39** * **(1.0–1.8)**	1.27 (0.9–1.7)	**1.38** * **(1.0–1.8)**	1.27 (0.9–1.7)	1.24 (0.9–1.6)	1.11 (0.8–1.5)	**1.48** ** **(1.1–2.0)**	1.22 (0.9–1.7)	**1.54** ** **(1.1–2.1)**	1.27 (0.9–1.7)
***Income Provider (at Birth)***										
Other	1.00	1.00	1.00	1.00	1.00	1.00	1.00	1.00	1.00	1.00
Mother	0.93 (0.6–1.5)	0.83 (0.5–1.4)	0.75 (0.5–1.2)	0.69 (0.4–1.2)	0.95 (0.6–1.5)	0.88 (0.5–1.5)	1.44 (0.9–2.3)	1.21 (0.7–2.1)	1.14 (0.7–1.9)	1.05 (0.6–1.9)
***Owns Fridge (at Birth)***										
Fridge: No	1.00	1.00	1.00	1.00	1.00	1.00	1.00	1.00	1.00	1.00
Fridge: Yes	1.19 (0.9–1.6)	1.01 (0.7–1.4)	**1.38** * **(1.0–1.8)**	1.14 (0.8–1.5)	1.28 (1.0–1.7)	1.16 (0.9–1.6)	1.28 (1.0–1.7)	1.10 (0.8–1.5)	**1.45** * **(1.1–1.9)**	1.18 (0.9–1.6)
***Perception of Wealth (Current)***										
Very comfortable	1.00	1.00	1.00	1.00	1.00	1.00	1.00	1.00	1.00	1.00
Getting by	0.63 (0.4–1.0)	0.70 (0.4–1.2)	0.85 (0.5–1.4)	0.91 (0.5–1.5)	0.98 (0.6–1.6)	1.08 (0.7–1.8)	1.05 (0.7–1.7)	1.13 (0.7–1.9)	0.81 (0.5–1.3)	0.81 (0.5–1.3)
Extremely poor	0.65 (0.4–1.1)	0.77 (0.4–1.3)	0.80 (0.5–1.3)	1.02 (0.6–1.8)	0.79 (0.5–1.3)	0.97 (0.6–1.7)	0.90 (0.5–1.5)	1.13 (0.7–1.9)	0.61 (0.4–1.0)	0.72 (0.4–1.3)
***Crèche***										
No crèche	1.00	1.00	1.00	1.00	1.00	1.00	1.00	1.00	1.00	1.00
Attended crèche	**2.38** ** **(1.4–4.2)**	**1.96** * **(1.1–3.5)**	1.57 (0.9–2.7)	1.14 (0.6–2.0)	1.17 (0.7–2.0)	0.99 (0.6–1.8)	1.21 (0.7–2.1)	1.00 (0.6–1.7)	1.22 (0.7–2.2)	0.87 (0.4–1.6)
***MC-HOME*** ^b^ ***(Current)***										
Low Total	1.00	1.00	1.00	1.00	1.00	1.00	1.00	1.00	1.00	1.00
High Total	1.06 (0.8–1.4)	0.93 (0.7–1.2)	1.22 (0.9–1.6)	1.12 (0.8–1.5)	1.29 (1.0–1.7)	1.19 (0.9–1.6)	1.11 (0.8–1.5)	1.07 (0.8–1.4)	1.02 (0.8–1.4)	0.94 (0.7–1.3)
***Maternal Mental Health (Current)***										
No mental disorders	1.00	1.00	1.00	1.00	1.00	1.00	1.00	1.00	1.00	1.00
Depression or anxiety or alcohol use	1.45 (0.9–2.3)	1.53 (0.9–2.6)	0.88 (0.5–1.4)	1.01 (0.6–1.7)	0.64 (0.4–1.1)	0.68 (0.4–1.2)	0.72 (0.4–1.2)	0.78 (0.5–1.3)	0.81 (0.5–1.4)	0.74 (0.4–1.3)
***Parenting Stress (Current)***										
Parenting stress ≤90	1.00	1.00	1.00	1.00	1.00	1.00	1.00	1.00	1.00	1.00
Parenting stress ≥90	0.91 (0.6–1.3)	0.97 (0.6–1.5)	**0.66** * **(0.4–1.0)**	0.74 (0.5–1.1)	0.74 (0.4–1.1)	0.82 (0.5–1.3)	0.75 (0.5–1.1)	0.83 (0.5–1.3)	0.99 (0.7–1.5)	1.14 (0.7–1.8)

When the association was significant, the odds ratio (OR) and adjusted odds ratio (aOR) are in bold (because of rounding, the confidence intervals sometimes overlap with 1.0). ORs are based on bivariate logistic regression of the outcome on the covariate. AORs are based on multivariate logistic regression on the outcomes including all covariates. Sequential Processing: measures audio and visual memory and memory span. Simultaneous Processing: measures spatial and visual perception, reasoning, and maths ability. Learning Ability: measures focused and selective attention and ability to store auditory and visual stimuli simultaneously. Planning: measures decision-making ability. Riddles, measures reasoning and language development. MC-HOME: Middle Childhood Home Observation for Measurement of the Environment.

^a^ High/low based on splitting the sample on the median.

^b^ High/low based on splitting the sample on the median.

* p ≤ 0.05;

** p < 0.01;

*** p < 0.001.

### Executive Function

None of the executive function measures were significantly associated with EBF duration, maternal HIV, or child sex (Table 3). Compared to children whose mothers were aged less than 20 y, those with mothers aged 20–29 y at their birth were almost twice as likely to score above the mean on the Animal Sorting subtest (aOR 1.82 [95% CI 1.2–2.8], *p* = 0.01), as were children whose mother was the main income provider during their infancy (aOR 1.81 [95% CI 1.0–3.1], *p* = 0.03) and those who had attended crèche (aOR 1.74 [95% CI 1.0–3.0], *p* = 0.05). For the Auditory Attention subtest, compared to children aged 8 y, those aged 9 and 10 y were over three (aOR 3.38 [95% CI 1.6–7.3], *p* = 0.01) and four times (aOR 4.56 [95% CI 2.1–9.9], *p* < 0.001) more likely to perform above average, respectively. Children with better stimulation at home (i.e., a HOME score above the median) were more likely to perform above the mean in the Auditory Attention (aOR 1.36 [95% CI 1.0–1.8], *p* = 0.04) and Response Set subtests (aOR 1.35 [95% CI 1.0–1.8], *p* = 0.05).

**Table 3:** Factors associated with children’s executive function measured by Developmental Neuropsychological Assessment (NEPSY-II).

	Animal Sorting (*n* = 824)		Auditory Attention (*n* = 821)		Response Set (*n* = 820)	
	OR (CI)	AOR (CI)	OR (CI)	AOR (CI)	OR (CI)	AOR (CI)
***Sex***						
Female	1.00	1.00	1.00	1.00	1.00	1.00
Male	0.93 (0.7–1.2)	0.93 (0.7–1.2)	0.83 (0.6–1.1)	0.83 (0.6–1.1)	0.92 (0.7–1.2)	0.97 (0.7–1.3)
***Child Age (Current)***						
8 y	1.00	1.00	1.00	1.00	1.00	1.00
9 y	0.63 (0.3–1.3)	0.70 (0.3–1.5)	**3.26** ** **(1.5–7.0)**	**3.38** ** **(1.6–7.3)**	1.77 (0.9–3.5)	1.60 (0.8–3.2)
10 y	0.55 (0.3–1.1)	0.65 (0.3–1.4)	**4.35** *** **(2.0–9.3)**	**4.56** *** **(2.1–9.9)**	1.55 (0.8–3.1)	1.39 (0.7–2.8)
11 y	0.72 (0.3–1.6)	0.87 (0.4–2.1)	1.83 (0.8–4.4)	1.98 (0.8–4.8)	0.79 (0.4–1.7)	0.73 (0.3–1.7)
***Mother’s Age (at Birth)***						
Less than 20 y	1.00	1.00	1.00	1.00	1.00	1.00
20–29 y	**1.98** *** **(1.4–2.9)**	**1.82** ** **(1.2–2.8)**	1.18 (0.8–1.7)	1.08 (0.7–1.7)	0.71 (0.5–1.1)	0.72 (0.5–1.1)
30+ y	**1.56** * **(1.1–2.3)**	1.60 (0.9–2.9)	**1.53** * **(1.0–2.3)**	1.53 (0.8–2.8)	**0.59** * **(0.4–0.9)**	0.69 (0.4–1.3)
***Maternal IQ (Current)*** ^a^						
Low Raven’s	1.00	1.00	1.00	1.00	1.00	1.00
High Raven’s	1.08 (0.8–1.4)	0.96 (0.7–1.3)	1.17 (0.9–1.5)	1.10 (0.8–1.5)	1.23 (0.9–1.6)	1.05 (0.8–1.4)
***Mother’s Education (at Birth)***						
None	1.00	1.00	1.00	1.00	1.00	1.00
Primary	1.12 (0.6–2.0)	1.09 (0.6–2.0)	0.56 (0.3–1.0)	0.62 (0.3–1.2)	1.06 (0.6–1.9)	1.02 (0.6–1.9)
Some secondary	1.27 (0.7–2.3)	1.23 (0.6–2.3)	0.75 (0.4–1.3)	0.99 (0.5–1.9)	1.61 (0.9–2.9)	1.47 (0.8–2.8)
Completed secondary/postsecondary	**1.87** * **(1.0–3.5)**	1.50 (0.7–3.0)	0.80 (0.4–1.5)	0.96 (0.5–2.0)	1.41 (0.8–2.6)	1.33 (0.7–2.7)
***Birthweight***						
Low birthweight	1.00	1.00	1.00	1.00	1.00	1.00
Normal birthweight	1.40 (0.9–2.2)	1.48 (0.9–2.5)	0.99 (0.6–1.6)	0.91 (0.6–1.4)	1.12 (0.7–1.8)	1.12 (0.7–1.8)
***Exclusive Breastfeeding***						
0–1 mo	1.00	1.00	1.00	1.00	1.00	1.00
2–5 mo	0.91 (0.6–1.5)	1.17 (0.7–1.9)	0.75 (0.5–1.2)	0.68 (0.4–1.1)	1.15 (0.7–1.9)	1.13 (0.7–1.9)
6 mo	0.98 (0.6–1.5)	1.35 (0.8–2.2)	0.78 (0.5–1.2)	0.69 (0.4–1.1)	1.08 (0.7–1.7)	1.09 (0.7–1.8)
***Birth Order (Birth)***						
Birth order 1–2	1.00	1.00	1.00	1.00	1.00	1.00
Birth order 3–4	1.13 (0.8–1.6)	0.92 (0.6–1.4)	1.15 (0.8–1.6)	1.14 (0.8–1.7)	0.78 (0.6–1.1)	0.98 (0.6–1.5)
Birth order 5+	0.90 (0.6–1.3)	0.77 (0.4–1.4)	1.21 (0.8–1.7)	1.11 (0.6–2.0)	**0.66** * **(0.5–0.9)**	0.94 (0.5–1.7)
***Mother’s HIV Status***						
Negative	1.00	1.00	1.00	1.00	1.00	1.00
Positive pregnancy	1.12 (0.8–1.5)	1.02 (0.7–1.4)	0.91 (0.7–1.2)	0.96 (0.7–1.4)	0.87 (0.6–1.2)	1.06 (0.8–1.5)
Positive since pregnancy	0.77 (0.5–1.1)	0.85 (0.6–1.3)	0.86 (0.6–1.2)	0.90 (0.6–1.4)	1.08 (0.7–1.6)	1.03 (0.7–1.6)
***Residence (at Birth)***						
Rural	1.00	1.00	1.00	1.00	1.00	1.00
Urban	**1.49** ** **(1.1–2.0)**	1.32 (1.0–1.8)	0.88 (0.7–1.2)	0.90 (0.7–1.2)	0.98 (0.7–1.3)	0.95 (0.7–1.3)
***Income Provider (at Birth)***						
Other	1.00	1.00	1.00	1.00	1.00	1.00
Mother	**1.84** * **(1.1–3.1)**	**1.81** * **(1.0–3.1)**	0.89 (0.5–1.5)	0.86 (0.5–1.5)	0.81 (0.5–1.3)	0.99 (0.6–1.7)
***Owns Fridge (at Birth)***						
Fridge: No	1.00	1.00	1.00	1.00	1.00	1.00
Fridge: Yes	1.29 (1.0–1.7)	1.19 (0.9–1.6)	1.21 (0.9–1.6)	1.19 (0.9–1.6)	1.02 (0.8–1.4)	1.05 (0.8–1.4)
***Perception of Wealth (Current)***						
Very comfortable	1.00	1.00	1.00	1.00	1.00	1.00
Getting by	0.97 (0.6–1.6)	1.14 (0.7–1.9)	1.17 (0.7–1.9)	1.20 (0.7–2.0)	1.05 (0.6–1.7)	1.09 (0.7–1.8)
Extremely poor	0.85 (0.5–1.4)	1.09 (0.6–1.9)	1.53 (0.9–2.6)	1.68 (1.0–2.9)	1.23 (0.7–2.1)	1.42 (0.8–2.5)
***Crèche***						
No crèche	1.00	1.00	1.00	1.00	1.00	1.00
Attended crèche	**1.85** * **(1.1–3.2)**	**1.74** * **(1.0–3.0)**	0.86 (0.5–1.5)	0.75 (0.4–1.3)	1.30 (0.8–2.2)	1.13 (0.6–2.0)
***MC-HOME*** ^b^ ***(Current)***						
Low Total	1.00	1.00	1.00	1.00	1.00	1.00
High Total	0.98 (0.7–1.3)	0.90 (0.7–1.2)	**1.39** * **(1.1–1.8)**	**1.36** * **(1.0–1.8)**	**1.35** * **(1.0–1.8)**	**1.35** * **(1.0–1.8)**
***Maternal Mental Health (Current)***						
No mental disorders	1.00	1.00	1.00	1.00	1.00	1.00
Depression or anxiety or alcohol use	1.00 (0.6–1.6)	1.02 (0.6–1.7)	1.04 (0.6–1.7)	1.07 (0.6–1.8)	1.05 (0.6–1.7)	1.05 (0.6–1.8)
***Parenting Stress (Current)***						
Parenting stress ≤90	1.00	1.00	1.00	1.00	1.00	1.00
Parenting stress ≥90	0.96 (0.6–1.4)	1.01 (0.7–1.6)	0.89 (0.6–1.3)	0.84 (0.5–1.3)	0.92 (0.6–1.4)	0.89 (0.6–1.4)

When the association was significant, the OR and aOR are in bold (because of rounding the confidence intervals sometimes overlap with 1.0). ORs are based on bivariate logistic regression of the outcome on the covariate. AORs are based on multivariate logistic regression on the outcomes including all covariates. Animal Sorting: measures inhibition, planning, cognitive flexibility. Auditory Attention: measures vigilance, selective/ sustained auditory attention. Response Set: measures inhibition of previously learned stimuli. MC-HOME: Middle Childhood HOME

^a^ High/low based on splitting the sample on the median.

^b^ High/low based on splitting the sample on the median.

* p ≤ 0.05;

** p < 0.01;

*** p < 0.001.

### Emotional and Behavioural Problems

Being born in an urban environment and having a primary caregiver with high parenting stress were associated with more emotional and behavioural problems (higher scores on the Internalising and Externalising subscales and the Total score) (urban Total score: aOR 1.62 [95% CI 1.2–2.2], *p* = 0.01; parenting stress Total score: aOR 7.04 [95% CI 4.2–11.9], *p* < 0.001). Children whose caregiver had a current mental health disorder were more likely to score above the mean for Internalising (aOR 1.92 [95% CI 1.1–3.4], *p* = 0.03) and Total scores (aOR 2.44 [95% CI 1.3–4.6], *p* = 0.01) (Table 4). Boys were more likely to score above the mean for Internalising (aOR 1.53 [95% CI 1.1–2.0], *p* = 0.01), whilst children who attended a crèche were approximately twice as likely to score above the mean in Externalising (aOR 2.15 [95% CI 1.2–3.9], *p* = 0.01) and Total scores (aOR 1.96 [95% CI 1.0–3.8], *p* = 0.05). EBF and the mother’s antenatal or current HIV status were not significantly associated with Externalising, Internalising, or Total CBCL score.

**Table 4:** Factors associated with children’s emotional and behavioural outcomes measured by the parent-reported Child Behaviour Checklist (CBCL).

	CBCL Internalising (*n* = 823)		CBCL Externalising (*n* = 823)		CBCL Total (*n* = 823)	
	OR (CI)	AOR (CI)	OR (CI)	AOR (CI)	OR (CI)	AOR (CI)
***Sex***						
Female	1.00	1.00	1.00	1.00	1.00	1.00
Male	**1.59** ** **(1.2–2.1)**	**1.53** ** **(1.1–2.0)**	1.13 (0.9–1.5)	1.03 (0.8–1.4)	**1.42** * **(1.1–1.9)**	1.33 (1.0–1.8)
***Child Age (Current)***						
8 y	1.00	1.00	1.00	1.00	1.00	1.00
9 y	0.87 (0.5–1.7)	0.81 (0.4–1.6)	1.00 (0.5–2.0)	0.97 (0.5–2.1)	1.04 (0.5–2.0)	1.04 (0.5–2.0)
10 y	0.74 (0.4–1.4)	0.70 (0.4–1.4)	0.90 (0.4–1.8)	0.97 (0.5–2.1)	0.90 (0.5–1.8)	0.99 (0.5–2.0)
11 y	0.66 (0.3–1.4)	0.72 (0.3–1.7)	0.99 (0.4–2.2)	1.35 (0.6–3.3)	0.98 (0.5–2.2)	1.38 (0.6–3.3)
***Mother’s Age (at Birth)***						
Less than 20 y	1.00	1.00	1.00	1.00	1.00	1.00
20–29 y	0.91 (0.6–1.3)	0.83 (0.5–1.3)	0.86 (0.6–1.2)	0.80 (0.5–1.2)	1.02 (0.7–1.5)	0.95 (0.6–1.5)
30+ y	0.88 (0.6–1.3)	0.90 (0.5–1.7)	0.84 (0.6–1.2)	0.85 (0.5–1.6)	0.99 (0.7–1.5)	1.15 (0.6–2.1)
***Maternal IQ (Current)*** ^a^						
Low Raven’s	1.00	1.00	1.00	1.00	1.00	1.00
High Raven’s	1.05 (0.8–1.4)	1.01 (0.7–1.4)	1.15 (0.9–1.5)	1.20 (0.9–1.7)	1.18 (0.9–1.6)	1.24 (0.9–1.7)
***Mother’s Education (at Birth)***						
None	1.00	1.00	1.00	1.00	1.00	1.00
Primary	1.24 (0.7–2.2)	1.11 (0.6–2.0)	1.12 (0.6–2.0)	1.02 (0.5–1.9)	1.06 (0.6–1.9)	0.89 (0.5–1.7)
Some secondary	1.23 (0.7–2.2)	1.08 (0.6–2.0)	1.18 (0.7–2.1)	0.93 (0.5–1.8)	1.13 (0.6–2.0)	0.83 (0.4–1.7)
Completed secondary/postsecondary	1.50 (0.8–2.8)	1.34 (0.7–2.7)	1.05 (0.6–2.0)	0.84 (0.4–1.7)	1.20 (0.6–2.2)	0.83 (0.4–1.8)
***Birthweight***						
Low birthweight	1.00	1.00	1.00	1.00	1.00	1.00
Normal birthweight	0.86 (0.5–1.3)	0.80 (0.5–1.3)	0.83 (0.5–1.3)	0.83 (0.5–1.4)	0.75 (0.5–1.2)	0.69 (0.4–1.1)
***Exclusive Breastfeeding***						
0–1 mo	1.00	1.00	1.00	1.00	1.00	1.00
2–5 mo	1.04 (0.7–1.7)	1.20 (0.7–2.0)	0.71 (0.4–1.2)	0.69 (0.4–1.2)	0.81 (0.5–1.3)	0.93 (0.6–1.6)
6 mo	0.94 (0.6–1.4)	1.12 (0.7–1.8)	0.81 (0.5–1.3)	0.81 (0.5–1.3)	0.75 (0.5–1.2)	0.87 (0.5–1.4)
***Birth Order (Birth)***						
Birth order 1–2	1.00	1.00	1.00	1.00	1.00	1.00
Birth order 3–4	0.96 (0.7–1.3)	0.97 (0.6–1.5)	0.96 (0.7–1.3)	0.98 (0.6–1.5)	0.93 (0.7–1.3)	0.86 (0.6–1.3)
Birth order 5+	0.83 (0.6–1.2)	0.83 (0.5–1.5)	0.85 (0.6–1.2)	0.81 (0.4–1.5)	0.80 (0.6–1.2)	0.66 (0.4–1.2)
***Mother’s HIV Status***						
Negative	1.00	1.00	1.00	1.00	1.00	1.00
Positive pregnancy	0.96 (0.7–1.3)	0.88 (0.6–1.3)	0.97 (0.7–1.3)	0.82 (0.6–1.2)	1.04 (0.8–1.4)	0.84 (0.6–1.2)
Positive since pregnancy	1.06 (0.7–1.5)	0.93 (0.6–1.4)	1.12 (0.8–1.6)	0.91 (0.6–1.4)	1.10 (0.8–1.6)	0.91 (0.6–1.4)
***Residence (at Birth)***						
Rural	1.00	1.00	1.00	1.00	1.00	1.00
Urban	**1.50** ** **(1.1–2.0)**	**1.45** * **(1.1–2.0)**	**1.35** * **(1.0–1.8)**	**1.39** * **(1.0–1.9)**	**1.56** ** **(1.2–2.1)**	**1.62** ** **(1.2–2.2)**
***Income Provider (at Birth)***						
Other	1.00	1.00	1.00	1.00	1.00	1.00
Mother	1.12 (0.7–1.8)	1.07 (0.6–1.8)	1.21 (0.7–2.0)	1.18 (0.7–2.1)	1.51 (0.9–2.4)	1.41 (0.8–2.4)
***Owns Fridge (at Birth)***						
Fridge: No	1.00	1.00	1.00	1.00	1.00	1.00
Fridge: Yes	1.07 (0.8–1.4)	1.07 (0.8–1.5)	1.00 (0.8–1.3)	0.99 (0.7–1.4)	1.01 (0.8–1.3)	0.94 (0.7–1.3)
***Perception of Wealth (Current)***						
Very comfortable	1.00	1.00	1.00	1.00	1.00	1.00
Getting by	0.96 (0.6–1.5)	1.04 (0.6–1.8)	1.07 (0.7–1.7)	1.18 (0.7–2.0)	0.68 (0.4–1.1)	0.74 (0.4–1.2)
Extremely poor	1.21 (0.7–2.0)	1.28 (0.7–2.3)	1.26 (0.8–2.1)	1.26 (0.7–2.2)	0.84 (0.5–1.4)	0.82 (0.5–1.5)
***Crèche***						
No crèche	1.00	1.00	1.00	1.00	1.00	1.00
Attended crèche	0.94 (0.6–1.6)	1.07 (0.6–1.9)	1.59 (0.9–2.7)	**2.15** * **(1.2–3.9)**	1.44 (0.8–2.5)	**1.96** * **(1.0–3.8)**
***MC-HOME*** ^b^ ***(Current)***						
Low Total	1.00	1.00	1.00	1.00	1.00	1.00
High Total	1.28 (1.0–1.7)	1.3 (1.0–1.7)	1.11 (0.8–1.5)	1.10 (0.8–1.5)	1.30 (1.0–1.7)	1.26 (0.9–1.7)
***Maternal Mental Health (Current)***						
No mental disorders	1.00	1.00	1.00	1.00	1.00	1.00
Depression or anxiety or alcohol use	**2.62** *** **(1.6–4.4)**	**1.92** * **(1.1–3.4)**	**2.29** ** **(1.4–3.8)**	1.58 (0.9–2.8)	**3.55** *** **(2.1–6.0)**	**2.44** ** **(1.3–4.6)**
***Parenting Stress (Current)***						
Parenting stress ≤90	1.00	1.00	1.00	1.00	1.00	1.00
Parenting stress ≥90	**3.79** *** **(2.4–6.0)**	**3.33** *** **(2.1–5.3)**	**5.75** *** **(3.6–9.2)**	**5.93** *** **(3.5–9.9)**	**7.22** *** **(4.4–11.8)**	**7.04** *** **(4.2–11.9)**

When the association was significant, the OR and aOR are in bold (because of rounding, the confidence intervals sometimes overlap with 1.0). ORs are based on bivariate logistic regression of the outcome on the covariate. AORs are based on multivariate logistic regression on the outcomes including all covariates

The CBCL includes 120-items in two subscales: “Internalising disorders” and “Externalising disorders,” and a composite Total score. A high score indicates more problems. MC-HOME: Middle Childhood HOME

^a^ High/low based on splitting the sample on the median.

^b^ High/low based on splitting the sample on the median.

* p ≤ 0.05;

** p < 0.01;

*** p < 0.001.

Exploring the six DSM disorders (Table 5), EBF was significantly associated with lower scores (fewer problems) for conduct disorders. Those who were exclusively breastfed for 6 mo compared to 1 mo or less were approximately half as likely to score above the mean for conduct disorders (aOR 0.44 [95% CI 0.3–0.7], *p* < 0.01). Caregiver mental health and stress were associated with increases in all six disorders. Urban residence was associated with increases in somatic, attention deficit hyperactivity disorder (ADHD), and oppositional problems. Boys were less likely to be anxious (aOR 0.64 [95% CI 0.5–0.9], *p* < 0.01) but more likely to have somatic (aOR 1.34 [95% CI 1.0–1.8], *p* = 0.05) or oppositional (aOR 1.52 [95% CI 1.1–2.2], *p* = 0.02) disorders.

**Table 5:** Child Diagnostic and Statistical Manual (DSM) Disorders measured by parent-reported CBCL (overall cohort of girls and boys).

	Affective (*n* = 823) AOR (CI)	Anxious (*n* = 823) AOR (CI)	Somatic (*n* = 823) AOR (CI)	ADHD (*n* = 823) AOR (CI)	Oppositional *(n* = 823) AOR (CI)	Conduct (*n* = 823) AOR (CI)
***Child Sex***						
Female	1.00	1.00	1.00	1.00	1.00	1.00
Male	1.09 (0.7–1.5)	**0.64** ** **(0.5–0.9)**	**1.34** * **(1.0–1.8)**	1.13 (0.8–1.6)	**1.52** * **(1.1–2.2)**	1.15 (0.8–1.6)
***Child Age (Current)***						
8 y	1.00	1.00	1.00	1.00	1.00	1.00
9 y	0.86 (0.4–2.0)	1.39 (0.6–3.1)	0.60 (0.3–1.2)	0.73 (0.3–1.7)	1.07 (0.5–2.4)	0.90 (0.4–2.1)
10 y	0.76 (0.3–1.9)	1.49 (0.7–3.4)	0.54 (0.3–1.1)	0.65 (0.3–1.5)	1.39 (0.6–3.2)	1.07 (0.5–2.5)
11 y	0.97 (0.3–2.8)	1.86 (0.7–4.7)	0.93 (0.4–2.1)	1.50 (0.5–4.2)	1.79 (0.6–5.0)	1.29 (0.5–3.5)
***Mother's Age (at Birth)***						
Less than 20 y	1.00	1.00	1.00	1.00	1.00	1.00
20–29 y	1.04 (0.6–1.7)	0.74 (0.5–1.2)	0.90 (0.6–1.4)	1.28 (0.8–2.2)	1.12 (0.7–1.9)	1.07 (0.7–1.7)
30+ y	1.17 (0.6–2.3)	0.73 (0.4–1.4)	0.98 (0.5–1.8)	0.99 (0.5–2.1)	1.17 (0.6–2.5)	1.07 (0.5–2.1)
***Maternal IQ (Current)*** ^a^						
Low Raven’s	1.00	1.00	1.00	1.00	1.00	1.00
High Raven’s	0.80 (0.6–1.2)	1.03 (0.7–1.4)	0.97 (0.7–1.3)	**1.49** * **(1.0–2.2)**	1.05 (0.7–1.5)	1.19 (0.8–1.7)
***Mother's Education (at Birth)***						
None	1.00	1.00	1.00	1.00	1.00	1.00
Primary	0.83 (0.4–1.8)	0.99 (0.5–1.8)	0.77 (0.4–1.4)	1.40 (0.7–3.0)	1.13 (0.5–2.5)	0.93 (0.5–1.9)
Some secondary	0.81 (0.4–1.8)	0.81 (0.4–1.6)	0.77 (0.4–1.5)	0.87 (0.4–2.0)	1.12 (0.5–2.6)	0.74 (0.3–1.6)
Completed secondary/postsecondary	0.49 (0.20–1.2)	0.91 (0.4–1.9)	0.82 (0.4–1.7)	0.61 (0.3–1.5)	1.25 (0.5–3.0)	**0.40** * **(0.1–0.9)**
***Birthweight***						
Low Birthweight	1.00	1.00	1.00	1.00	1.00	1.00
Normal Birthweight	0.66 (0.4–1.1)	0.70 (0.4–1.1)	0.88 (0.6–1.4)	0.65 (0.4–1.1)	0.68 (0.4–1.2)	0.72 (0.4–1.2)
***Exclusive Breastfeeding***						
0–1 mo	1.00	1.00	1.00	1.00	1.00	1.00
2–5 mo	0.94 (0.5–1.7)	0.92 (0.5–1.6)	1.18 (0.7–2.0)	0.81 (0.4–1.5)	0.76 (0.4–1.4)	**0.35** *** **(0.2–0.6)**
6 mo	0.94 (0.5–1.7)	0.90 (0.5–1.5)	1.11 (0.7–1.8)	0.94 (0.5–1.6)	0.89 (0.5–1.6)	**0.44** ** **(0.3–0.7)**
***Birth Order (Birth)***						
Birth order 1–2	1.00	1.00	1.00	1.00	1.00	1.00
Birth order 3–4	0.64 (0.4–1.1)	0.84 (0.5–1.3)	1.00 (0.7–1.5)	0.65 (0.4–1.0)	0.85 (0.5–1.4)	0.71 (0.4–1.1)
Birth order 5+	0.54 (0.3–1.1)	0.80 (0.4–1.5)	0.93 (0.5–1.7)	0.67 (0.3–1.4)	0.78 (0.8–1.6)	**0.44** * **(0.2–0.9)**
***Mother's HIV Status***						
Negative	1.00	1.00	1.00	1.00	1.00	1.00
Positive pregnancy	0.97 (0.6–1.5)	0.90 (0.6–1.3)	0.86 (0.6–1.2)	0.86 (0.6–1.3)	**0.58** * **(0.4–0.9)**	0.74 (0.5–1.1)
Positive since pregnancy	1.32 (0.8–2.1)	1.01 (0.7–1.5)	0.75 (0.5–1.1)	1.00 (0.6–1.6)	0.70 (0.4–1.2)	0.68 (0.4–1.1)
***Residence (at Birth)***						
Rural	1.00	1.00	1.00	1.00	1.00	1.00
Urban	1.13 (0.8–1.7)	1.21 (0.9–1.7)	**1.46** * **(1.0–2.0)**	1.44 (1.0–2.1)	**1.57** * **(1.1–2.3)**	1.26 (0.9–1.8)
***Income Provider (at Birth)***						
Other	1.00	1.00	1.00	1.00	1.00	1.00
Mother	0.73 (0.4–1.4)	0.93 (0.5–1.7)	1.06 (0.6–1.8)	1.66 (0.9–3.0)	0.80 (0.4–1.6)	0.98 (0.5–1.9)
***Owns Fridge (at Birth)***						
Fridge: No	1.00	1.00	1.00	1.00	1.00	1.00
Fridge: Yes	0.81 (06–1.2)	0.92 (0.7–1.3)	0.82 (0.6–1.1)	1.04 (0.7–1.5)	0.91 (0.6–1.3)	1.01 (0.7–1.4)
***Perception of Wealth (Current)***						
Very comfortable	1.00	1.00	1.00	1.00	1.00	1.00
Getting by	1.15 (0.6–2.2)	0.93 (0.5–1.6)	1.20 (0.7–2.0)	0.65 (0.4–1.2)	0.68 (0.4–1.2)	0.89 (0.5–1.6)
Extremely poor	1.38 (0.7–2.8)	1.55 (0.9–2.8)	1.22 (0.7–2.2)	0.88 (0.5–1.7)	0.75 (0.4–1.4)	1.02 (0.5–1.9)
***Crèche***						
No crèche	1.00	1.00	1.00	1.00	1.00	1.00
Attended crèche	1.25 (0.6–2.5)	0.98 (0.5–1.8)	1.01 (0.6–1.8)	1.97 (0.9–4.3)	**3.42** ** **(1.5–8.0)**	1.34 (0.7–2.6)
***MC-HOME*** ^b^ ***(Current)***						
Low Total	1.00	1.00	1.00	1.00	1.00	1.00
High Total	**1.54** * **(1.1–2.2)**	1.24 (0.9–1.7)	1.26 (0.9–1.7)	0.79 (0.6–1.1)	1.17 (0.8–1.7)	0.87 (0.6–1.2)
***Maternal Mental Health (Current)***						
No mental disorders	1.00	1.00	1.00	1.00	1.00	1.00
Depression or anxiety or alcohol use	**1.97** * **(1.1–3.6)**	**2.03** ** **(1.2–3.4)**	**1.93** * **(1.1–3.3)**	1.40 (0.8–2.6)	**1.95** * **(1.1–3.6)**	1.77 (1.0–3.2)
***Parenting Stress (Current)***						
Parenting stress ≤90	1.00	1.00	1.00	1.00	1.00	1.00
Parenting stress ≥90	**5.39** *** **(3.4–8.5)**	**2.23** *** **(1.4–3.5)**	**2.08** ** **(1.4–3.2)**	**4.85** *** **(3.1–7.7)**	**4.68** *** **(3.0–7.4)**	**5.80** *** **(3.7–9.1)**

When the association was significant, the OR and aOR are in bold (because of rounding, the confidence intervals sometimes overlap with 1.0). ORs are based on bivariate logistic regression of the outcome on the covariate. AORs are based on multivariate logistic regression on the outcomes including all covariates. Shown in the table are the CBCL scores for the six DSM disorders: affective, anxious, somatic, attention deficit hyperactivity, oppositional, and conduct disorders. MC-HOME: Middle Childhood HOME.

^a^ High/low based on splitting the sample on the median.

^b^ High/low based on splitting the sample on the median.

* p ≤ 0.05;

** p < 0.01;

*** p < 0.001.

### Outcomes Stratified by Sex (Tables 6–9)

**Table 6:** Girls: Factors associated with children's cognitive and executive function outcomes measured by the KABC-II and NEPSY-II.

	Cognitive Assessment					Executive Function Assessment		
	Sequential	Planning	Learning	Simultaneous	Riddles	Animal Sorting	Auditory Attention	Response Set
	AOR (CI)	AOR (CI)	AOR (CI)	AOR (CI)	AOR (CI)	AOR (CI)	AOR (CI)	AOR (CI)
***Child Age (Current)***								
8 y	1	1	1	1	1	1	1	1
9 y	0.94 (0.4–2.3)	0.83 (0.4–1.8)	0.85 (0.4–2.0)	0.65 (0.3–1.6)	2.13 (0.8–5.5)	0.77 (0.3–2.0)	2.23 (0.9–5.9)	2.21 (0.8–5.9)
10 y	0.82 (0.3–2.1)	1.02 (0.4–2.3)	0.95 (0.4–2.3)	0.43 (0.2–1.1)	1.54 (0.6–4.1)	0.68 (0.2–1.9)	**4.00** ** **(1.5–10.8)**	2.64 (1.0–7.2)
11 y	0.95 (0.3–3.0)	0.94 (0.3–2.8)	1.04 (0.3–3.2)	0.74 (0.2–2.2)	0.91 (0.3–3.1)	0.99 (0.3–3.4)	1.84 (0.6–5.8)	0.77 (0.2–2.5)
***Mother's Age (at Birth)***								
Less than 20 y	1	1	1	1	1	1	1	1
20–29 y	0.99 (0.6–1.8)	0.77 (0.4–1.4)	0.97 (0.5–1.7)	1.25 (0.7–2.2)	1.07 (0.6–2.0)	1.51 (0.8–2.7)	1.78 (1.0–3.2)	**0.46** * **(0.2–0.9)**
30+ y	1.67 (0.7–3.8)	0.94 (0.4–2.1)	2.17 (1.0–4.9)	1.06 (0.5–2.4)	1.42 (0.6–3.3)	0.92 (0.4–2.1)	2.01 (0.9–4.6)	0.54 (0.2–1.4)
***Maternal IQ (Current)*** ^a^								
Low Raven’s	1	1	1	1	1	1	1	1
High Raven’s	1.15 (0.7–1.8)	1.07 (0.7–1.7)	1.31 (0.8–2.0)	0.96 (0.6–1.5)	1.23 (0.8–1.9)	0.88 (0.6–1.4)	1.06 (0.7–1.7)	1.05 (0.7–1.7)
***Mother's Education (at Birth)***								
None	1	1	1	1	1	1	1	1
Primary	0.79 (0.4–1.7)	0.85 (0.4–1.9)	1.15 (0.5–2.7)	1.54 (0.7–3.5)	0.73 (0.3–1.7)	0.93 (0.4–2.0)	0.64 (0.3–1.5)	0.76 (0.3–1.7)
Some secondary	0.99 (0.4–2.2)	2.02 (0.9–4.7)	1.45 (0.6–3.5)	**2.65** * **(1.1–6.3)**	1.36 (0.6–3.3)	1.03 (0.5–2.3)	1.42 (0.6–3.3)	1.98 (0.8–4.8)
Completed secondary/postsecondary	1.36 (0.5–3.4)	2.39 (0.9–6.2)	2.02 (0.8–5.3)	**4.10** ** **(1.6–10.8)**	2.47 (0.9–6.5)	1.72 (0.7–4.4)	0.91 (0.4–2.4)	1.23 (0.5–3.3)
***Birthweight***								
Low Birthweight	1	1	1	1	1	1	1	1
Normal Birthweight	1.39 (0.7–2.7)	**2.04** * **(1.1–3.9)**	**2.40** * **(1.2–5.0)**	1.82 (0.9–3.5)	1.17 (0.5–2.6)	1.42 (0.7–2.8)	1.13 (0.6–2.1)	1.36 (0.7–2.7)
***Exclusive Breastfeeding***								
0–1 mo	1	1	1	1	1	1	1	1
2–5 mo	1.34 (0.6–2.8)	0.70 (0.3–1.5)	0.57 (0.3–1.2)	1.10 (0.5–2.3)	1 (0.5–2.2)	1.16 (0.5–2.6)	**0.35** ** **(0.2–0.8)**	1.06 (0.5–2.4)
6 mo	1.69 (0.8–3.5)	0.49 (0.2–1.0)	0.77 (0.4–1.6)	1.07 (0.5–2.2)	0.90 (0.4–2.0)	1.53 (0.7–3.3)	**0.46** * **(0.2–1.0)**	1.15 (0.5–2.6)
***Birth Order (Birth)***								
Birth order 1–2	1	1	1	1	1	1	1	1
Birth order 3–4	0.74 (0.4–1.3)	0.97 (0.5–1.7)	0.75 (0.4–1.3)	1.25 (0.7–2.2)	1.13 (0.6–2.1)	1.23 (0.7–2.2)	1.01 (0.6–1.8)	1.37 (0.8–2.5)
Birth order 5+	0.62 (0.3–1.4)	1.17 (0.51–2.6)	**0.40** * **(0.2–0.9)**	1.47 (0.6–3.4)	0.98 (0.4–2.3)	1.42 (0.6–3.2)	1.61 (0.7–3.8)	0.98 (0.4–2.4)
***Mother's HIV Status***								
Negative	1	1	1	1	1	1	1	1
Positive pregnancy	0.96 (0.6–1.6)	**0.53** * **(0.3–0.9)**	1.05 (0.6–1.7)	0.71 (0.4–1.1)	0.89 (0.5–1.5)	0.93 (0.6–1.5)	0.86 (0.5–1.4)	0.93 (0.6–1.6)
Positive since pregnancy	0.79 (0.4–1.4)	0.75 (0.4–1.4)	0.82 (0.5–1.5)	0.63 (0.3–1.2)	1.13 (0.6–2.0)	0.97 (0.6–1.7)	0.90 (0.5–1.6)	0.86 (0.4–1.6)
***Residence (at Birth)***								
Rural	1	1	1	1	1	1	1	1
Urban	1.41 (0.9–2.2)	1.48 (0.9–2.3)	1.10 (0.7–1.7)	1.26 (0.8–2.0)	1.59 (1.0–2.5)	1.29 (0.8–2.0)	0.85 (0.5–1.4)	0.98 (0.6–1.6)
***Income Provider (at Birth)***								
Other	1	1	1	1	1	1	1	1
Mother	0.93 (0.4–2.1)	0.78 (0.3–1.8)	1.07 (0.5–2.4)	1.26 (0.5–2.9)	1.30 (0.5–3.3)	1.57 (0.7–3.6)	0.45 (0.2–1.1)	0.94 (0.4–2.2)
***Owns Fridge (at Birth)***								
Fridge: No	1	1	1	1	1	1	1	1
Fridge: Yes	1.11 (0.7–1.7)	1.37 (0.9–2.1)	1.35 (0.9–2.1)	1.46 (0.9–2.3)	0.96 (0.7–1.5)	1.09 (0.7–1.7)	1.25 (0.8–2.0)	1.52 (1.0–2.4)
***Perception of Wealth (Current)***								
Very comfortable	1	1	1	1	1	1	1	1
Getting by	0.52 (0.2–1.0)	0.81 (0.4–1.7)	1.43 (0.7–3.0)	1.44 (0.7–3.0)	0.70 (0.3–1.4)	0.99 (0.5–2.1)	1.03 (0.5–2.2)	1.07 (0.5–2.2)
Extremely poor	0.61 (0.3–1.4)	1.09 (0.4–2.4)	1.18 (0.5–2.6)	1.17 (0.5–2.6)	0.64(0.3–1.4)	0.73 (0.3–1.6)	1.06 (0.5–2.4)	1.43 (0.7–3.1)
***Crèche***								
No crèche	1	1	1	1	1	1	1	1
Attended crèche	1.17 (0.5–3.0)	1.04 (0.4–2.6)	1.01 (0.4–2.9)	0.75 (0.3–2.0)	0.66 (0.2–1.8)	2.47 (0.9–6.5)	0.42 (0.2–1.1)	2.03 (0.7–5.9)
***MC-HOME*** ^b^ ***(Current)***								
Low Total	1	1	1	1	1	1	1	1
High Total	0.91 (0.6–1.4)	0.95 (0.6–1.5)	1.01 (0.6–1.6)	0.89 (0.6–1.4)	0.78 (0.5–1.2)	0.85 (0.6–1.3)	**1.73** * **(1.1–2.7)**	**1.69** * **(1.1–2.6)**
***Maternal Mental Health (Current)***								
No mental disorders	1	1	1	1	1	1	1	1
Depression or anxiety or alcohol use	2.08 (0.9–4.6)	1.19 (0.6–2.5)	0.60 (0.3–1.3)	0.91 (0.4–2.0)	0.68 (0.3–1.5)	0.72 (0.3–1.6)	1.18 (0.5–2.6)	2.12 (0.9–4.7)
***Parenting Stress (Current)***								
Parenting stress ≤90	1	1	1	1	1	1	1	1
Parenting stress ≥90	0.72 (0.4–1.4)	0.76 (0.4–1.5)	1.12 (0.5–2.3)	1.08 (0.5–2.2)	1.51 (0.8–3.0)	1.31 (0.6–2.7)	0.64 (0.3–1.2)	0.66 (0.3–1.3)

When the association was significant, the OR and aOR are in bold (because of rounding, the confidence intervals sometimes overlap with 1.0). AORs are based on multivariate logistic regression on the outcomes including all covariates. Sequential Processing: measures audio and visual memory and memory span. Simultaneous Processing: measures spatial and visual perception, reasoning, and maths ability. Learning Ability: measures focused and selective attention and ability to store auditory and visual stimuli simultaneously. Planning: measures decision-making ability. Riddles: measures reasoning and language development. Animal Sorting: measures inhibition, planning, and cognitive flexibility. Auditory Attention: measures vigilance and selective/sustained auditory attention. Response Set: measures inhibition of previously learned stimuli. MC-HOME: Middle Childhood HOME

^a^ High/low based on splitting the sample on the median.

^b^ High/low based on splitting the sample on the median.

* p ≤ 0.05;

** p < 0.01;

*** p < 0.001.

**Table 7:** Boys: Factors associated with children's cognitive and executive function outcomes measured by the KABC-II and NEPSY-II.

	Cognitive Assessment					Executive Function Assessment		
	Sequential	Planning	Learning	Simultaneous	Riddles	Animal Sorting	Auditory Attention	Response Set
	AOR (CI)	AOR (CI)	AOR (CI)	AOR (CI)	AOR (CI)	AOR (CI)	AOR (CI)	AOR (CI)
***Child Age (Current)***								
8 y	1.00	1.00	1.00	1.00	1.00	1.00	1.00	1.00
9 y	1.32 (0.5–3.9)	2.42 (0.8–7.5)	1.56 (0.5–4.9)	0.68 (0.2–2.5)	0.37 (0.1–1.2)	0.55 (0.2–1.8)	**12.69** * **(1.7–95.3)**	0.93 (0.3–2.8)
10 y	1.02 (0.3–3.0)	1.87 (0.6–5.9)	1.16 (0.4–3.7)	0.56 (0.2–2.1)	**0.27** * **(0.1–0.9)**	0.57 (0.2–1.9)	**14.46** * **(1.9–109.1)**	0.62 (0.2–1.9)
11 y	0.77 (0.2–2.8)	0.77 (0.2–3.0)	1.25 (0.3–4.8)	0.37 (0.1–1.6)	**0.14** ** **(0.0–0.6)**	0.93 (0.2–3.7)	**6.74** * **(0.8–56.9)**	0.53 (0.1–1.9)
***Mother's Age (at Birth)***								
Less than 20 y	1.00	1.00	1.00	1.00	1.00	1.00	1.00	1.00
20–29 y	1.16 (0.6–2.2)	0.83 (0.4–1.6)	1.37 (0.7–2.6)	1.05 (0.6–1.9)	1.07 (0.5–2.1)	**2.17** * **(1.1–4.1)**	0.60 (0.3–1.1)	1.20 (0.6–2.3)
30+ y	1.54 (0.6–3.8)	0.62 (0.2–1.6)	1.61 (0.7–3.8)	1.39 (0.6–3.3)	0.92 (0.3–2.6)	**2.89** * **(1.1–7.40)**	0.97 (0.4–2.3)	0.96 (0.4–2.2)
***Maternal IQ (Current)*** ^a^								
Low Raven’s	1.00	1.00	1.00	1.00	1.00	1.00	1.00	1.00
High Raven’s	**1.77** * **(1.1–2.8)**	**2.79** *** **(1.8–4.4)**	**2.05** ** **(1.3–3.2)**	**2.19** ** **(1.4–3.5)**	**2.20** ** **(1.4–3.6)**	1.00 (0.6–1.6)	1.06 (0.7–1.7)	1.04 (0.7–1.6)
***Mother's Education (at Birth)***								
None	1.00	1.00	1.00	1.00	1.00	1.00	1.00	1.00
Primary	1.24 (0.5–3.4)	1.30 (0.5–3.3)	0.71 (0.3–1.9)	1.13 (0.4–2.9)	1.19 (0.3–4.1)	1.25 (0.5–3.3)	0.43 (0.1–1.3)	1.29 (0.5–3.4)
Some secondary	1.51 (0.5–4.4)	1.03 (0.4–2.8)	0.72 (0.3–2.0)	0.74 (0.3–2.1)	1.57 (0.4–5.6)	1.64 (0.6–4.6)	0.55 (0.2–1.7)	1.30 (0.5–3.7)
Completed secondary/postsecondary	1.72 (0.5–5.4)	1.31 (0.4–4.0)	0.96 (0.3–2.9)	1.25 (0.4–3.7)	1.25 (0.3–4.8)	1.47 (0.5–4.5)	0.84 (0.3–2.8)	1.52 (0.5–4.6)
***Birthweight***								
Low birthweight	1.00	1.00	1.00	1.00	1.00	1.00	1.00	1.00
Normal birthweight	1.15 (0.5–2.4)	0.97 (0.4–2.1)	0.69 (0.3–1.4)	1.09 (0.5–2.3)	2.29 (0.8–6.2)	1.68 (0.8–3.7)	0.77 (0.4–1.6)	0.93 (0.5–1.9)
***Exclusive Breastfeeding***								
0–1 mo	1.00	1.00	1.00	1.00	1.00	1.00	1.00	1.00
2–5 mo	1.24 (0.6–2.5)	1.08 (0.5–2.2)	**2.07** * **(1.0–4.3)**	1.52 (0.7–3.2)	1.12 (0.5–2.4)	1.14 (0.6–2.3)	1.12 (0.6–2.2)	1.27 (0.6–2.5)
6 mo	0.90 (0.5–1.7)	1.13 (0.6–2.1)	1.87 (0.9–3.7)	1.59 (0.8–3.1)	1.58 (0.8–3.1)	1.17 (0.6–2.3)	0.85 (0.5–1.6)	1.06 (0.6–2.0)
***Birth Order (Birth)***								
Birth order 1–2	1.00	1.00	1.00	1.00	1.00	1.00	1.00	1.00
Birth order 3–4	1.03 (0.6–1.9)	1.31 (0.7–2.5)	0.97 (0.5–1.8)	0.86 (0.5–1.6)	1.45 (0.7–2.9)	0.69 (0.4–1.3)	1.46 (0.8–2.7)	0.74 (0.4–1.4)
Birth order 5+	0.84 (0.4–2.0)	1.89 (0.8–4.5)	0.99 (0.4–2.3)	1.21 (0.5–2.9)	1.23 (0.4–3.4)	**0.40** * **(0.2–1.0)**	0.91 (0.4–2.1)	0.94 (0.4–2.1)
***Mother's HIV Status***								
Negative	1.00	1.00	1.00	1.00	1.00	1.00	1.00	1.00
Positive pregnancy	1.27 (0.8–2.1)	1.09 (0.7–1.8)	1.26 (0.7–2.2)	1.16 (0.7–1.9)	**1.92** * **(1.1–3.3)**	1.20 (0.7–2.0)	1.02 (0.6–1.7)	1.00 (0.6–1.7)
Positive since pregnancy	0.84 (0.5–1.5)	**0.55** * **(0.3–1.0)**	1.57 (0.9–2.8)	0.71 (0.4–1.3)	0.88 (0.5–1.7)	0.75 (0.4–1.4)	0.78 (0.4–1.4)	1.11 (0.6–2.0)
***Residence (at Birth)***								
Rural	1.00	1.00	1.00	1.00	1.00	1.00	1.00	1.00
Urban	1.14 (0.7–1.8)	1.10 (0.7–1.7)	1.12 (0.7–1.8)	1.17 (0.7–1.8)	0.91 (0.6–1.5)	1.27 (0.8–2.0)	0.95 (0.6–1.5)	0.99 (0.6–1.6)
***Income Provider (at Birth)***								
Other	1.00	1.00	1.00	1.00	1.00	1.00	1.00	1.00
Mother	0.75 (0.4–1.5)	0.62 (0.3–1.2)	0.72 (0.3.–1.5)	1.36 (0.7–2.8)	0.87 (0.4–1.9)	1.96 (1.0–4.0)	1.43 (0.7–3.0)	1.03 (0.5–2.1)
***Owns Fridge***								
Fridge: No	1.00	1.00	1.00	1.00	1.00	1.00	1.00	1.00
Fridge: Yes	0.95 (0.6–1.5)	0.97 (0.6–1.5)	1.04 (0.7–1.6)	0.87 (0.6–1.4)	1.56 (1.0–2.5)	1.31 (0.8–2.1)	1.32 (0.8–2.1)	0.74 (0.5–1.2)
***Perception of Wealth (Current)***								
Very comfortable	1.00	1.00	1.00	1.00	1.00	1.00	1.00	1.00
Getting by	0.96 (0.4–2.1)	0.83 (0.4–1.8)	0.77 (0.4–1.5)	0.72 (0.3–1.6)	0.88 (0.4–1.9)	1.40 (0.7–2.9)	1.49 (0.7–3.1)	1.09 (0.5–2.2)
Extremely poor	1.00 (0.4–2.3)	0.72 (0.3–1.6)	0.74 (0.3–1.5)	0.82 (0.3–1.9)	0.70 (0.3–1.6)	1.69 (0.7–3.7)	**2.81** * **(1.2–6.3)**	1.41 (0.6–3.1)
***Crèche***								
No crèche	1.00	1.00	1.00	1.00	1.00	1.00	1.00	1.00
Attended crèche	**2.74** ** **(1.3–5.9)**	1.23 (0.6–2.6)	0.91 (0.5–1.8)	1.12 (0.6–2.2)	0.93 (0.4–2.0)	1.73 (0.9–3.5)	0.97 (0.5–2.0)	0.91 (0.5–1.8)
***MC-HOME*** ^b^ ***(Current)***								
Low Total	1.00	1.00	1.00	1.00	1.00	1.00	1.00	1.00
High Total	0.96 (0.6–1.5)	1.32 (0.9–2.0)	1.39 (0.9–2.1)	1.37 (0.9–2.1)	1.00 (0.6–1.6)	0.84 (0.5–1.3)	1.08 (0.7–1.7)	1.11 (0.7–1.7)
***Maternal Mental Health (Current)***								
No mental disorders	1.00	1.00	1.00	1.00	1.00	1.00	1.00	1.00
Depression or anxiety or alcohol use	1.32 (0.6–2.9)	0.94 (0.4–2.1)	0.71 (0.3–1.5)	0.64 (0.3–1.3)	0.79 (0.4–1.8)	1.44 (0.7–2.8)	1.03 (0.5–2.2)	0.55 (0.3–1.1)
***Parenting Stress (Current)***								
Parenting stress ≤90	1.00	1.00	1.00	1.00	1.00	1.00	1.00	1.00
Parenting stress ≥90	1.14 (0.7–1.9)	0.73 (0.4–1.3)	0.67 (0.4–1.2)	0.71 (0.4–1.2)	0.96 (0.5–1.8)	0.90 (0.50–1.6)	1.00 (0.6–1.7)	0.95 (0.6–1.6)

When the association was significant, the OR and aOR are in bold (because of rounding, the confidence intervals sometimes overlap with 1.0). AORs are based on multivariate logistic regression on the outcomes including all covariates. Sequential Processing: measures audio and visual memory and memory span. Simultaneous Processing: measures spatial and visual perception, reasoning, and maths ability. Learning Ability: measures focused and selective attention and ability to store auditory and visual stimuli simultaneously. Planning: measures decision-making ability. Riddles: measures reasoning and language development. Animal Sorting: measures inhibition, planning, and cognitive flexibility. Auditory Attention: measures vigilance and selective/sustained auditory attention. Response Set: measures inhibition of previously learned stimuli. MC-HOME: Middle Childhood HOME

^a^ High/low based on splitting the sample on the median.

^b^ High/low based on splitting the sample on the median.

* p ≤ 0.05;

** p < 0.01;

*** p < 0.001.

**Table 8:** Girls: Factors associated with children's emotional and behavioural outcomes measured by the parent-reported CBCL.

	CBCL Internalising	CBCL Externalising	CBCL Total
	AOR (CI)	AOR (CI)	AOR (CI)
***Child Age (Current)***			
8 y	1	1	1
9 y	1.14 (0.5–2.9)	1.20 (0.4–3.3)	1.52 (0.6–4.2)
10 y	0.95 (0.4–2.5)	1.33 (0.5–3.8)	1.52 (0.5–4.3)
11 y	0.99 (0.3–3.2)	1.80 (0.5–6.2)	1.98 (0.6–7.0)
***Mother's Age (at Birth)***			
Less than 20 y	1	1	1
20–29 y	0.69 (0.4–1.3)	0.88 (0.5–1.6)	0.81 (0.4–1.5)
30+ y	1.21 (0.5–2.9)	1.50 (0.6–3.5)	1.40 (0.6–3.3)
***Maternal IQ (Current)*** ^a^			
Low Raven’s	1	1	1
High Raven’s	0.93 (0.6–1.5)	1.39 (0.9–2.2)	1.21 (0.8–1.9)
***Mother's Education (at Birth)***			
None	1	1	1
Primary	1.37 (0.6–3.3)	1.42 (0.6–3.3)	1.99 (0.7–5.6)
Some secondary	1.23 (0.5–3.0)	1.22 (0.5–3.0)	1.96 (0.7–5.7)
Completed secondary/postsecondary	1.90 (0.7–5.1)	1.51 (0.6–4.1)	2.70 (0.9–8.6)
***Birthweight***			
Low birthweight	1	1	1
Normal birthweight	0.66 (0.4–1.2)	0.62 (0.3–1.2)	**0.42** * **(0.2–0.9)**
***Exclusive Breastfeeding***			
0–1 mo	1	1	1
2–5 mo	1.95 (0.9–4.4)	1.13 (0.5–2.5)	1.51 (0.7–3.4)
6 mo	1.96 (0.9–4.3)	1.37 (0.6–3.0)	1.35 (0.6–3.0)
***Birth Order (Birth)***			
Birth order 1–2	1	1	1
Birth order 3–4	0.92 (0.5–1.7)	0.85 (0.5–1.6)	0.84 (0.4–1.6)
Birth order 5+	0.74 (0.3–1.8)	**0.38** * **(0.2–0.9)**	0.49 (0.2–1.2)
***Mother's HIV Status***			
Negative	1	1	1
Positive pregnancy	0.80 (0.5–1.3)	**0.56** * **(0.2–0.9)**	**0.56** * **(0.3–1.0)**
Positive since pregnancy	0.73 (0.4–1.3)	0.73 (0.4–1.3)	0.73 (0.4–1.4)
***Residence (at Birth)***			
Rural	1	1	1
Urban	1.49 (0.9–2.4)	**1.61** * **(1.0–2.6)**	**2.00** ** **(1.2–3.2)**
***Income Provider (at Birth)***			
Other	1	1	1
Mother	0.72 (0.3–1.6)	1.55 (0.7–3.6)	1.22 (0.5–2.8)
***Owns Fridge (at Birth)***			
Fridge: No	1	1	1
Fridge: Yes	0.99 (0.6–1.5)	1.21 (0.8–1.9)	1.04 (0.7–1.7)
***Perception of Wealth (Current)***			
Very comfortable	1	1	1
Getting by	0.70 (0.3–1.5)	1.36 (0.7–2.8)	0.57 (0.3–1.2)
Extremely poor	1.16 (0.5–2.6)	2.11 (0.9–4.7)	0.83 (0.4–1.9)
***Crèche***			
No crèche	1	1	1
Attended crèche	3.78 (1.0–14.8)	1.40 (0.5–4.0)	1.84 (0.5–6.5)
***MC-HOME*** ^b^ ***(Current)***			
Low Total	1	1	1
High Total	1.30 (0.8–2.0)	1.05 (0.7–1.6)	1.17 (0.7–1.8)
***Maternal Mental Health (Current)***			
No mental disorders	1	1	1
Depression or anxiety or alcohol use	**3.24** ** **(1.3–7.8)**	**2.98** ** **(1.3–6.7)**	**3.28** ** **(1.4–8.0)**
***Parenting Stress (Current)***			
Parenting stress ≤90	1	1	1
Parenting stress ≥90	**3.25** ** **(1.5–6.9)**	**2.44** * **(1.2–5.2)**	**4.63** *** **(2.1–10.1)**

When the association was significant, the OR and aOR are in bold (because of rounding, the confidence intervals sometimes overlap with 1.0.). AORs are based on multivariate logistic regression on the outcomes including all covariates. The CBCL includes 120-items in two subscales: “Internalising Disorders” and “Externalising Disorders,” and a composite Total score. A high score indicates more problems. MC-HOME: Middle Childhood HOME

^a^ High/low based on splitting the sample on the median.

^b^ High/low based on splitting the sample on the median.

* p ≤ 0.05;

** p < 0.01;

*** p < 0.001.

**Table 9:** Boys: Factors associated with children's emotional and behavioural outcomes measured by the parent-reported CBCL.

	CBCL Internalising	CBCL Externalising	CBCL Total
	AOR (CI)	AOR (CI)	AOR (CI)
***Child Age (Current)***			
8 y	1.00	1.00	1.00
9 y	0.30 (0.1–1.2)	0.76 (0.2–2.5)	0.64 (0.2–2.1)
10 y	**0.26** * **(0.1–1.0)**	0.62 (0.2–2.1)	0.56 (0.2–1.8)
11 y	0.37 (0.1–1.7)	0.99 (0.2–4.1)	1.07 (0.3–4.4)
***Mother's Age (at Birth)***			
Less than 20 y	1.00	1.00	1.00
20–29 y	0.93 (0.5–1.8)	0.82 (0.4–1.6)	1.25 (0.6–2.4)
30+ y	0.66 (0.3–1.6)	0.52 (0.2–1.3)	1.11 (0.4–2.8)
***Maternal IQ (Current)*** ^a^			
Low Raven’s	1.00	1.00	1.00
High Raven’s	1.21 (0.8–1.9)	1.07 (0.7–1.8)	1.36 (0.8–2.2)
***Mother's Education (at Birth)***			
None	1.00	1.00	1.00
Primary	0.73 (0.3–1.8)	0.63 (0.2–1.6)	**0.30** * **(0.1–0.8)**
Some secondary	0.82 (0.3–2.2)	0.60 (0.2–1.7)	**0.26** * **(0.1–0.7)**
Completed secondary/postsecondary	0.82 (0.3–2.4)	0.42 (0.1–1.2)	**0.21** ** **(0.1–0.6)**
***Birthweight***			
Low birthweight	1.00	1.00	1.00
Normal birthweight	0.93 (0.4–2.0)	1.06 (0.5–2.3)	1.21 (0.6–2.6)
***Exclusive Breastfeeding***			
0–1 mo	1.00	1.00	1.00
2–5 mo	0.84 (0.4–1.8)	**0.48** * **(0.2–1.0)**	0.66 (0.3–1.4)
6 mo	0.68 (0.3–1.3)	0.56 (0.3–1.1)	0.63 (0.3–1.3)
***Birth Order (Birth)***			
Birth order 1–2	1.00	1.00	1.00
Birth order 3–4	1.06 (0.6–2.0)	1.10 (0.6–2.1)	0.81 (0.4–1.5)
Birth order 5+	0.83 (0.4–2.0)	1.36 (0.6–3.3)	0.62 (0.3–1.4)
***Mother's HIV Status***			
Negative	1.00	1.00	1.00
Positive pregnancy	0.94 (0.6–1.5)	1.10 (0.6–1.9)	1.15 (0.7–2.0)
Positive since pregnancy	1.28 (0.7–2.4)	1.09 (0.6–2.1)	1.13 (0.6–2.2)
***Residence (at Birth)***			
Rural	1.00	1.00	1.00
Urban	1.40 (0.9–2.3)	1.18 (0.7–1.9)	1.31 (0.8–2.1)
***Income Provider (at Birth)***			
Other	1.00	1.00	1.00
Mother	1.55 (0.7–3.3)	1.07 (0.5–2.4)	1.75 (0.8–3.8)
***Owns Fridge (at Birth)***			
Fridge: No	1.00	1.00	1.00
Fridge: Yes	1.30 (0.8–2.1)	0.93 (0.6–1.5)	0.98 (0.6–1.6)
***Perception of Wealth (Current)***			
Very comfortable	1.00	1.00	1.00
Getting by	1.78 (0.8–4.2)	1.06 (0.5–2.3)	0.96 (0.4–2.1)
Extremely poor	1.65 (0.7–4.2)	0.81 (0.3–1.9)	0.82 (0.3–1.9)
***Crèche***			
No crèche	1.00	1.00	1.00
Attended crèche	0.63 (0.3–1.3)	**2.55** * **(1.2–5.2)**	2.14 (1.0–4.7)
***MC-HOME*** ^b^ ***(Current)***			
Low Total	1.00	1.00	1.00
High Total	1.26 (0.8–2.0)	1.19 (0.8–1.9)	1.36 (0.9–2.1)
***Maternal Mental Health (Current)***			
No mental disorders	1.00	1.00	1.00
Depression or anxiety or alcohol use	1.48 (0.7–3.4)	1.31 (0.6–3.0)	**2.63** * **(1.0–7.0)**
***Parenting Stress (Current)***			
Parenting stress ≤90	1.00	1.00	1.00
Parenting stress ≥90	**3.46** *** **(1.8–6.6)**	**10.35** *** **(4.9–21.9)**	**9.72** *** **(4.6–20.6)**

When the association was significant, the OR and aOR are in bold (because of rounding, the confidence intervals sometimes overlap with 1.0). AORs are based on multivariate logistic regression on the outcomes including all covariates. The CBCL includes 120-items in two subscales: “Internalising Disorders” and “Externalising Disorders,” and a composite Total score. A high score indicates more problems. MC-HOME: Middle Childhood HOME

^a^ High/low based on splitting the sample on the median.

^b^ High/low based on splitting the sample on the median.

* p ≤ 0.05;

** p < 0.01;

*** p < 0.001.

Contrary to the finding in the overall cohort, boys, but not girls, who were exclusively breastfed for more than 1 mo were twice as likely as those who were exclusively breastfed for a very short period to score above the mean for Learning Ability (aOR 2.07 [95% CI 1.0–4.3], *p* = 0.05) and half as likely to score above the mean for Externalising problems (aOR 0.48 [95% CI 0.2–1.0], *p* = 0.05) (Tables 7 and 9). However, girls who were exclusively breastfed for less than 1 mo were more likely to score above average on Auditory Attention compared to those who were exclusively breastfed longer. The finding of an association between maternal cognitive ability and improved performance on all four cognitive subscales in the overall cohort held for boys but not for girls. Boys whose mother’s cognitive ability was above the median score (Standard Raven’s Progressive Matrix) were twice as likely to score above average for the cognitive subscales (e.g., Planning subscale aOR 2.79 [95% CI 1.8–4.4], *p* ≤ 0.001). Maternal HIV status was not significantly associated with cognitive development, executive function, or emotional-behavioural problems overall. However, boys whose mothers became infected with HIV after pregnancy were more likely to score below the mean on the Planning Ability subscale than those whose mothers remained HIV negative (aOR 0.55 [95% CI 0.3–1.0], *p* = 0.05). Boys born to HIV-positive mothers, compared to those born to HIV-negative mothers, were more likely to score above average for reasoning and language ability (Riddles subtest) (aOR 1.92 [95% CI 1.1–3.3], *p* = 0.02). For girls, being born to an HIV-positive mother was associated with scoring below the mean for Planning Ability (aOR 0.53 [95% CI 0.3–0.9], *p* = 0.01) and below the mean (fewer problems) for Externalising (aOR 0.56 [95% CI 0.2–0.9], *p* = 0.03) and Total (aOR 0.56 [95% CI 0.3–1.0], *p* = 0.03) CBCL scores.

Girls, but not boys, with a normal birthweight were significantly more likely to score above the mean for Learning Ability (aOR 2.40 [95% CI 1.2–5.0], *p* = 0.02) and Planning Ability (aOR 2.04 [95% CI 1.1–3.9], *p* = 0.03) and below the mean (fewer problems) for Total CBCL score (aOR 0.42 [95% CI 0.2–0.9], *p* = 0.02). Boys of a birth order of five or more were less likely than firstborns to score above average on Animal Sorting (inhibition, planning, and cognitive flexibility) (aOR 0.40 [95% CI 0.2–1.0], *p* = 0.04); girls of a birth order of five or more were significantly more likely to have a lower Externalising CBCL score (aOR 0.38 [95% CI 0.2–0.9], *p* = 0.03).

In this stratified analysis, the associations between maternal education and cognitive outcomes and HOME scores and executive function held for girls but not for boys. Likewise, girls were approximately three times more likely to score above the median for higher emotional-behavioural problems if their mothers had a mental health problem (Total CBCL aOR 3.28 [95% CI 1.4–8.0], *p* = 0.01) or high parenting stress (Total CBCL 4.63 [2.1–10.1], *p* < 0.001).

In the full model, interactions between sex and EBF were significant for Conduct (*p* = 0.02) and CBCL Internalising (*p* = 0.02) outcomes and marginally significant for Learning (*p* = 0.07) and Auditory Attention (*p* = 0.08).

In summary, while duration of EBF was not associated with child cognitive development or executive function in the overall sample (including girls and boys), it was associated with fewer conduct disorders, and, when stratified by sex, there was weak evidence of improved cognitive development in boys.

## Discussion

To our knowledge, this is the first study examining EBF, HIV-exposure, and child cognition, executive function, and emotional-behavioural outcomes at primary school age in Africa.

Our finding that duration of EBF was associated with fewer conduct disorders has significant implications. Conduct disorders lead to aggressive or disruptive behaviours that interfere with learning and peer relationships, in turn leading to low self-esteem and further behavioural problems [39]. Conduct disorders in childhood are associated with an increase in violent criminal behaviour and poor long-term mental health and academic achievement in later life [40]. While to our knowledge there have been no formal analyses of the economic costs of conduct disorders in low-middle-income countries, the evidence from carefully conducted studies in high-income countries is that the costs are enormous [41]. For example, a report from the United Kingdom stated that the total cost of crime attributable to people who had a conduct disorder in childhood was estimated to be £60 billion per annum [42]. Given these costs to individuals, families, and society, it is highly relevant that this study has shown EBF to be associated with reduced likelihood of conduct disorders at this critical stage of development. Further, for boys, a longer EBF duration was weakly associated with a doubling of the odds of better cognitive development on the Learning subscale, which assesses ability for maintaining focused attention while coding and storing complex auditory and visual stimuli simultaneously and generating strategies to learn efficiently. Identifying potential strategies to improve the life chances of boys is important, and, in our context, EBF appears to be associated with a longer-term advantage for boys. There is increasing evidence that some groups of children are more susceptible to the effects of negative rearing and, importantly, that they may benefit more from the effects of positive rearing and interventions. This is known as the “differential susceptibility hypothesis” [42]. Boys may be more susceptible in a number of domains of development to the effects of negative rearing—for example, in language development [43,44]—and therefore, in line with our findings, may benefit more from early EBF.

The finding that EBF did not have an effect on children’s cognitive development overall is in accord with studies from other resource-limited settings that also found no association [4], including studies in India (*n* = 514, aged 9–10 y), China (*n* = 442, aged 3 y), Malaysia (*n* = 1,394, aged 9 y), Chile (*n* = 784, aged 5.5 y), and the Philippines (*n* = 1,984, aged 8.5 and 11.5 y), although these studies did not stratify by sex.

An important strength of this study is the investigation of a wide range of key determinants of child development. In our cohort, maternal cognitive ability was strongly positively associated with children’s performance across all cognitive subscales, but not with executive function, although higher maternal cognitive ability was associated with improved performance on the Planning subscale, which assesses ability to focus attention, make decisions, and apply working memory, abilities considered to reflect executive function [27]. Interestingly, while having attended a crèche was associated with improvement in children’s cognitive development (sequential scores that depend on practice, on which boys scored particularly low) at the primary school age, it was independently associated with poorer behaviour, with similar associations found for children born in urban and rural settings. This apparent paradox in relation to crèche exposure is consistent with data from high-income countries [45].

Executive function is a high-order cognitive function that coordinates and controls information processing [18]. It is critical in enabling a child to successfully integrate into environments such as school [46]. We found a positive association between executive function and better stimulation at home, older maternal age, and a mother who was the main income provider at the time of the child’s birth. These findings are in accord with evidence suggesting that executive function is susceptible to environmental factors [47]. Executive function is increasingly thought of as the core ingredients (creativity, flexibility, self-control, and discipline) that will determine a child’s success in life. There is evidence that executive function is negatively affected by stress, emotions, poor physical fitness, and childhood obesity [39]. Encouragingly, a growing body of evidence suggests that simple, low-cost interventions such as noncomputerized games, aerobics, martial arts, and mindfulness may support improvements in executive function [19].

Our finding that poor maternal mental health and high parenting stress, measured at the time of the child assessments, were associated with increased emotional-behavioural problems in children was unsurprising but useful to quantify in an African setting. The link between parental mental health and child behaviour outcomes is well-established, with children of depressed, anxious, or highly stressed mothers known to be at increased risk of psychological and behavioural problems [48,49].

There is a dearth of evidence examining the developmental trajectories of HIV-exposed children, in particular longitudinal studies including HIV-unexposed controls. A recent systematic review [26] examining HIV exposure and child development found only 11 studies (1,591 children aged 0–18 y) and showed that HIV-exposed children are disadvantaged compared to their HIV-unexposed peers, in particular in emotional-behavioural and, to a lesser extent, cognitive development. However, results are not consistent across research settings or age groups, with most of the current evidence being based on small samples with wide heterogeneity in outcome measures [26]. Our results, based on a large sample of children including HIV-unexposed controls suggest that, overall, HIV-exposed children performed as well as HIV-unexposed children in the domains examined and that the findings of other small studies may be overstated.

Limitations include the nonrandomised study design of the original intervention, the lack of maternal mental health measurement at the child’s birth, and no assessment of cognition or emotional/behavioural problems at earlier time points in the children’s lives. Infections of the developing brain and childhood malnutrition also affect later cognitive ability but were not included in the analyses. In addition, caution must be taken in interpreting the sex-stratified models, as examining subgroup effects increases uncertainty and is more likely to produce larger effect estimates. Further, because of the complexities in measuring cognitive and executive function and the need to model each of the outcomes separately, multiple tests were used, and there may be some false positive significant results. However, our results are in line with findings from other studies.

Strengths include a large cohort of HIV-exposed and HIV-unexposed children, population normal values, adjustment for a wide range of confounders including current maternal IQ, and a battery of culturally appropriate developmental assessments including executive function and behavioural outcomes. A unique strength in this study is the accurate documentation of daily EBF data. Both HIV-positive and HIV-negative women had high rates of EBF, with few breast health problems, likely due to the quality of lay breastfeeding counselling [25,50]. Given that all women received intensive support, it is possible that this may have limited our ability to detect differences between EBF and non-EBF children.

In conclusion, while EBF was not significantly associated with cognitive development at the primary-school age, there was an association between EBF and a reduction in conduct disorders and, for boys, weak evidence of positive associations both in aspects of cognitive development and behavioural problems more generally. Given the number of adverse factors in these families’ environments, including poverty and high HIV prevalence, the fact that these benefits were evident into the crucial early school years is important. Child outcomes were associated with a range of other key factors. While core cognitive development was principally associated with maternal cognitive abilities, executive function was associated with a number of modifiable environmental factors including home stimulation and crèche attendance. Child’s emotional development was largely associated with caregiver mental health. These findings highlight a number of avenues for potential interventions.

## Supporting Information

S1 STROBE Checklist Checklist of items that should be included in reports of observational studies. (DOCX)

S1 Table Factors associated with children's cognitive and executive function continuous outcomes measured by the KABC-II and NEPSY-II. (DOCX)

## Abbreviations

ADHD: attention deficit hyperactivity disorder
aOR: adjusted odds ratio
AUDIT-6: Alcohol Use Disorders Identification Test
BREC: Biomedical Research Ethics Committee
CBCL: Child Behaviour Checklist
DSM: Diagnostic and Statistical Manual
DSS: Demographic Surveillance System
EBF: exclusive breastfeeding
GAD-7 scale: Generalized Anxiety Disorder 7-item scale
HOME: Home Observation for Measurement of the Environment
IQ: intelligence quotient
KABC-II: Kaufman Assessment Battery for Children
MC-HOME: Middle Childhood HOME
NEPSY-II: Developmental Neuropsychological Assessment Second Edition
OR: odds ratio
PHQ-9: Patient Health Questionnaire Depression
PSI-36: Parenting Stress Index Short Form
VTS: Vertical Transmission Study
WHO: World Health Organization

## References

[1] World Health Organization, UNICEF. Global strategy for infant and young child feeding. 2003.

[2] Bhandari N, Bahl R, Mazumdar S, Martines J, Black RE, Bhan MK, et al Effect of community-based promotion of exclusive breastfeeding on diarrhoeal illness and growth: a cluster randomised controlled trial. Lancet. 2003;361(9367):1418–23. 12727395 10.1016/S0140-6736(03)13134-0

[3] Lamberti LM, Walker CLF, Noiman A, Victora C, Black RE. Breastfeeding and the risk for diarrhea morbidity and mortality. BMC Public Health. 2011;11(Suppl 3):S15 10.1186/1471-2458-11-S3-S15 21501432 PMC3231888

[4] Walfisch A, Sermer C, Cressman A, Koren G. Breast milk and cognitive development—the role of confounders: a systematic review. BMJ Open. 2013;3(8). 10.1136/bmjopen-2013-003259 PMC3753522 23975102

[5] Horta BL, Victora CG. Long-term effects of breastfeeding. Geneva, Switzerland:: 2013.

[6] Quigley MA, Hockley C, Carson C, Kelly Y, Renfrew MJ, Sacker A. Breastfeeding is associated with improved child cognitive development: A population-based cohort study. J Pediatr. 2012;160(1):25–32. 10.1016/j.jpeds.2011.06.035 21839469

[7] Belfort MB, Rifas-Shiman SL, Kleinman KP, Guthrie LB, Bellinger DC, Taveras EM, et al Infant feeding and childhood cognition at ages 3 and 7 years: effects of breastfeeding duration and exclusivity. JAMA Pediatr. 2013;167(9):836–44. 23896931 10.1001/jamapediatrics.2013.455 PMC3998659

[8] Oddy WH, Li J, Whitehouse AJO, Zubrick SR, Malacova E. Breastfeeding duration and academic achievement at 10 years. Pediatrics. 2011;127(1):e137–e45. 10.1542/peds.2009-3489 21172993

[9] Kramer MS, Aboud F, Mironova E, Vanilovich I, Platt RW, Matush L, et al Breastfeeding and child cognitive development: new evidence from a large randomized trial. Arch Gen Psychiatry. 2008;65(5):578–84. 10.1001/archpsyc.65.5.578 18458209

[10] Victora CG, Horta BL, de Mola CL, Quevedo L, Pinheiro RT, Gigante DP, et al Association between breastfeeding and intelligence, educational attainment, and income at 30 years of age: a prospective birth cohort study from Brazil. Lancet Glob Health. 2015;3(4):e199–e205. 10.1016/S2214-109X(15)70002-1 25794674 PMC4365917

[11] Greiner T. Exclusive breastfeeding: measurement and indicators. Int Breastfeed J 2014;9(1):18. 25349624 10.1186/1746-4358-9-18 PMC4209171

[12] Bland R, Rollins N, Solarsh G, Van den Broeck J, Coovadia H. Maternal recall of exclusive breast feeding duration. Arch Dis Child. 2003;88(9):778–83. 12937095 10.1136/adc.88.9.778 PMC1719625

[13] Florey CdV, Leech A, Blackhall A. Infant feeding and mental and motor development at 18 months of age in first born singletons. Int J Epidemiol. 1995;24(Supplement 1):S21–S6. 7558546 10.1093/ije/24.supplement_1.s21

[14] Sloan S, Stewart M, Dunne L. The effect of breastfeeding and stimulation in the home on cognitive development in one-year-old infants. Child Care in Practice. 2010;16(2):101–10.

[15] Thorsdottir I, Gunnarsdóttir I, Kvaran M, Gretarsson S. Maternal body mass index, duration of exclusive breastfeeding and children's developmental status at the age of 6 years. Eur J Clin Nutr. 2005;59(3):426–31. 15674314 10.1038/sj.ejcn.1602092

[16] Nassar MF, Younis NT, El-Arab SE, Fawzi FA. Neuro-developmental outcome and brain-derived neurotrophic factor level in relation to feeding practice in early infancy. Matern Child Nutr. 2011;7(2):188–97. 10.1111/j.1740-8709.2010.00252.x 21410884 PMC6860701

[17] Diamond A. Executive functions. Annu Rev Psychol. 2013;64:135–68. 10.1146/annurev-psych-113011-143750 23020641 PMC4084861

[18] Obradović J, Portilla XA, Boyce WT. Executive functioning and developmental neuroscience: Current progress and implications for early childhood education In: Pianta RC, Justice L, Barnett S, Sheridan S, editors. The Handbook of Early Education. New York, NY: Guilford press; 2012 p. 324–51.

[19] Diamond A, Lee K. Interventions shown to aid executive function development in children 4 to 12 years old. Science. 2011;333(6045):959–64. 10.1126/science.1204529 21852486 PMC3159917

[20] Bland R, Coovadia H, Coutsoudis A, Rollins N, Newell M. Cohort profile: mamanengane or the Africa centre vertical transmission study. Int J Epidemiol. 2010;39(2):351–60. 10.1093/ije/dyp165 19336438 PMC2846440

[21] Coovadia HM, Rollins NC, Bland RM, Little K, Coutsoudis A, Bennish ML, et al Mother-to-child transmission of HIV-1 infection during exclusive breastfeeding in the first 6 months of life: an intervention cohort study. Lancet. 2007;369(9567):1107–16. 17398310 10.1016/S0140-6736(07)60283-9

[22] Patel D, Bland R, Coovadia H, Rollins N, Coutsoudis A, Newell ML. Breastfeeding, HIV status and weights in South African children: a comparison of HIV-exposed and unexposed children. AIDS. 2010;24(3):437–45. 10.1097/QAD.0b013e3283345f91 19915445

[23] Rollins NC, Ndirangu J, Bland RM, Coutsoudis A, Coovadia HM, Newell ML. Exclusive breastfeeding, diarrhoeal morbidity and all-cause mortality in infants of HIV-infected and HIV uninfected mothers: an intervention cohort study in KwaZulu Natal, South Africa. PLoS ONE. 2013;8(12):e81307 10.1371/journal.pone.0081307 24312545 PMC3846835

[24] Tanser F, Hosegood V, Barnighausen T, Herbst K, Nyirenda M, Muhwava W, et al Cohort Profile: Africa Centre Demographic Information System (ACDIS) and population-based HIV survey. Int J Epidemiol. 2008;37(5):956–62. 17998242 10.1093/ije/dym211 PMC2557060

[25] Bland RM, Little KE, Coovadia HM, Coutsoudis A, Rollins NC, Newell M-L. Intervention to promote exclusive breast-feeding for the first 6 months of life in a high HIV prevalence area. AIDS. 2008;22(7):883–91. 10.1097/QAD.0b013e3282f768de 18427207

[26] Sherr L, Croome N, Castaneda KP, Bradshaw K. A systematic review of psychological functioning of children exposed to HIV: Using evidence to plan for tomorrow’s HIV needs. AIDS Behav. 2014;18(11):2059–74. 10.1007/s10461-014-0747-6 24729015

[27] Kaufman AS, Kaufman NL. KABC-II Kaufman Assessment Battery for Children. Bloomington, MN, United States: Pearson; 2004.

[28] Bangirana P, Seggane M, Allebeck P, Giordani B, John CC, Opoka OR, et al A preliminary examination of the construct validity of the KABC-II in Ugandan children with a history of cerebral malaria. Afri Health Sci. 2009;9(3):186–92. PMC2887024 20589149

[29] Korkman M, Kirk U, Kemp S. NEPSY-II Neuropsychological Assessment Battery Bloomington, MN, United States: Pearson; 2007.

[30] Achenbach TM, Rescorla LA. Manual for the ASEBA School-Age Forms & Profiles Burlington, VT: University of Vermont, Research Center for Children, Youth, & Families; 2001.

[31] Achenbach TM, Rescorla LA. Multicultural Supplement to the Manual for the ASEBA School-Age Forms & Profiles. Burlington, VT: University of Vermont, Research Center for Children, Youth, & Families; 2007.

[32] Kroenke K, Spitzer RL, Williams JBW. Instruction Maunal—Patient Health Questionaire (PHQ) and GAD-7 Measures Patient Health Questionaire (PHQ) Screeners: Pfizer; 2002 http://www.phqscreeners.com/.

[33] Kroenke K, Spitzer RL, Williams JBW, Löwe B. The Patient Health Questionnaire Somatic, Anxiety, and Depressive Symptom Scales: a systematic review. Gen Hosp Psychiatry. 2010;32(4):345–59. 10.1016/j.genhosppsych.2010.03.006 20633738

[34] Babor TF, Higgins-Biddlle JC, Saunders JB, Monteiro MG. AUDIT The Alcohol Use Disoders Identification Test 2nd Edition: Guidelines for primary care. Geneva: Department of Mental Health and Substance Dependence, World Health Organisation; 2001.

[35] Abidin R. Parenting Stress Index Professional Manual (3rd Edition). Florida, United States: Psychological Assessement Resources Incorporated 1995.

[36] Caldwell B, Bradely R. Home Observation for Measurement of the Environment (HOME)-revised edition. Little Rock, AR: University of Arkansas, Little Rock; 1984.

[37] Raven J, Raven JC, Court JH. Raven's Standard Progressive Matrices (SPM) Manual 2000 Edition. Bloomington, United States: Pearsons; 2004.

[38] Halpern DF. Sex differences in cognitive abilities: Psychology Press; 2013.

[39] Masten AS, Roisman GI, Long JD, Burt KB, Obradović J, Riley JR, et al Developmental cascades: linking academic achievement and externalizing and internalizing symptoms over 20 years. Dev Psychol. 2005;41(5):733 16173871 10.1037/0012-1649.41.5.733

[40] Pilling S, Gould N, Whittington C, Taylor C, Scott S. Recognition, intervention, and management of antisocial behaviour and conduct disorders in children and young people: summary of NICE-SCIE guidance. BMJ. 2013;346. 10.1136/bmj.f1298 23535256

[41] NICE. Antisocial behaviour and conduct disorders in children and young people: recognition, intervention and management’ NICE Clinical Guideline 158 2013;Costing Report.

[42] Belsky J, Bakermans-Kranenburg MJ, Van IJzendoorn MH. For better and for worse differential susceptibility to environmental influences. Curr Dir Psychol Sci. 2007;16(6):300–4.

[43] Bornstein MH, Hahn C-S, Haynes OM. Specific and general language performance across early childhood: Stability and gender considerations. First Lang 2004;24(3):267–304.

[44] Bornstein MH. Children's Parents. Handbook of Child Psychology and Developmental Science Ecological Settings and Processes. New York: John Wiley & Sons, Inc; 2015 p. 1–78.

[45] Belsky J, Vandell DL, Burchinal M, Clarke-Stewart KA, McCartney K, Owen MT, et al Are there long-term effects of early child care? Child Dev. 2007;78(2):681–701. 17381797 10.1111/j.1467-8624.2007.01021.x

[46] Portilla XA, Ballard PJ, Adler NE, Boyce WT, Obradović J. An integrative view of school functioning: Transactions between self‐regulation, school engagement, and teacher–child relationship quality. Child Dev. 2014;85(5):1915–31. 10.1111/cdev.12259 24916608 PMC4165700

[47] Fay-Stammbach T, Hawes DJ, Meredith P. Parenting Influences on Executive Function in Early Childhood: A Review. Child Dev Perspect. 2014;8(4):258–64.

[48] Stein A, Desmond C, Garbarino J, van IJzendoorn MH, Barbarin O, Black MM, et al Predicting long-term outcomes for children affected by HIV and AIDS: perspectives from the scientific study of children's development. AIDS. 2014;28:S261–S8. 10.1097/QAD.0000000000000328 24991899 PMC10875626

[49] Goodman S, Rouse M, Connell A, Broth M, Hall C, Heyward D. Maternal Depression and Child Psychopathology: A Meta-Analytic Review. Clin Child Fam Psychol Rev. 2011;14(1):1–27. 10.1007/s10567-010-0080-1 21052833

[50] Bland R, Becquet R, Rollins N, Coutsoudis A, Coovadia H, Newell ML. Breast health problems are rare in both HIV-infected and uninfected women who receive counseling and support for breast-feeding in South Africa. Clin Infect Dis. 2007;45:1502–10. 17990235 10.1086/523320

