# Supplementary material for: Exclusive Breastfeeding and Cognition, Executive Function, and Behavioural Disorders in Primary School-Aged Children in Rural South Africa: A Cohort Analysis
Source: PLoS Med. 2016 Jun 21;13(6):e1002044. doi: 10.1371/journal.pmed.1002044 (PMC4915617; doi:10.1371/journal.pmed.1002044)
Supplement: S1 STROBE Checklist — (DOCX) [file pmed.1002044.s001.docx]

STROBE Statement

Checklist of items that should be included in reports of observational studies

# Section: Title and abstract

1. **Recommendations for manuscript title and abstract have been met, specifically:**
   1. Title makes clear the study design “a cohort analysis”
   2. Abstract provides a balanced and informative summary

# Section: Introduction

1. **We have explained the scientific rational and background for the investigations being reported as follows:**

See section head “Introduction”

Paragraph 1 outlines the existing scientific evidence relating to EBF and children’s development and highlight limitations in that evidence, including: the lack of evidence from low resourced settings; small sample sizes; lack of rigour in documentation of breastfeeding patterns; and inconsistency between studies.

Paragraph 1, last sentence, outlines that the population under study (HIV exposed and unexposed children in rural Africa) are a highly vulnerable and under-researched group, of considerable interest to policy makers.

Paragraph 2 outlines the methodological limitations in the current literature around child development assessments. We highlight the importance of adequate measures of cognitive development and executive function in the examination of children’s later outcomes.

Paragraph 3 outlines the cohort under investigation in this manuscript, providing a reference to the study registration number on clinicaltrial.gov and indicating why this cohort is particularly suited to answering the questions addressed by this research.

1. **We have stated the objectives and a proposed hypothesis**

See section head “Introduction”

Paragraph 3 line 6 clearly states the objectives of the investigation.

Paragraph 3 Line 10 we include our hypothesis, and line 8-11 outlines for the reader the important confounders we have controlled for in testing this hypothesis.

# Section: Methods

1. **Key elements of the study design are identified early in the paper, specifically:**

See section head “Methods”

Paragraph 1 indicates that this is a “a non-randomised, prospective, intervention cohort study”

1. **We have described the setting of the research including:**

See section head “Methods”

Paragraph 1 line 2 provides information on the study location and the relevant dates of the original VTS intervention study along with the time frame of the original VTS intervention.

Paragraph 1 line 5 provides the dates for the re-enrolment for the developmental assessments.

In paragraph 10, sub section heading “Data collection” we provide the recruitment dates of the current follow up and a clear description of the data collection process.

1. **Participants are clearly described, since this is a cohort study, in line with the checklist requirements we state:**

In the section head “Methods” we address the following issues:

**Eligibility:** paragraph 3 lists the eligibility criteria.

**Sources and methods of selection**: Paragraph 2 addresses the sources and methods of selection of VTS participants; describe sources and methods of selection for DSS participants.

**Matching criteria**: in paragraph 2 we also describe the matching criteria for the children included from the DSS to establish the population mean.

**Methods of follow up**: In paragraph 10 under the sub heading “Data collection” we describe the method of follow up in detail.

**Number of exposed/unexposed to intervention**: In paragraph 2, line 2 we identify the numbers of children who are unexposed to intervention (i.e. DSS children); paragraph 2, line 8 identifies the numbers of children who are exposed to intervention (i.e. VTS children).

Further, **Figure 1** presents a consort diagram to illustrate and support the above points.

1. **We have clearly defined all outcomes, exposures, predictors, potential confounders and effect moderators and given diagnostic information where application**

In the section head “Methods” we address the following issues:

Paragraph 1 line 3-4 we describe all exposure variables.

Paragraph 4, 5, 6, 7, 8 and 9 under the subheading “Outcome measures” defines all outcome measures, maternal and child variables, and potential confounders.

Paragraph 12, under the subheading “Statistical analysis” we describe the main exposure variable, EBF, and how it was measured in detail.

In Table 1, as a footnote we provide diagnostic information for maternal mental health measures.

1. **We have described all data sources and measures:**

In the section head “Methods”

Paragraph 2 lines 1-2 describe data sources for both the exposed (to intervention, i.e. VTS) and unexposed children (i.e. DSS) as per checklist requirements.

In the section head “Methods” under the subheading “Data collection”

Paragraph 1 describes the data collection methods and clarifies for the reader that all current measures are equivalent amongst exposed and unexposed groups.

In the section head “Methods”

Paragraph 3, line 1 identifies for the reader where data is prospectively, and in line 5 retrospectively, collected.

1. **Describe any efforts to address potential sources of bias**

In the section head “Methods” under the subheading “Statistical analysis”

Paragraph 1 line 18-26; and Table 1 addresses potential bias.

In the section head “Methods”

Paragraph 3 we outline the strength of this analysis, the rigorous data on daily breastfeeding data.

Under the section head “Discussion” paragraph 8 outlines the limitation of the study.

1. **Explain how the study size was arrived at**

In the section head “Methods” in paragraph 2 we describe the sample size calculation.

1. **Explain how quantitative variables were handled in the analysis**

In the section head “Methods” under the subheading “Statistical analysis”

Paragraph 1 explains in detail how each of the quantitative variables was addressed in the analyses. The use of the population mean to strengthen this analysis is also explained.

1. **Describe statistical methods**

In the section head “Methods” under the subheading “Statistical analysis” we note the following for readers:

- 1. Controlling for confounding (see paragraph 1 lines 15-16)
  2. Subgroups and interactions (see paragraph 1 lines 18-19)
  3. Explain how missing data were addressed (see paragraph 2)
  4. Loss to follow up and clear reasons for such within the VTS group are presented in **Figure 1**
  5. Sensitivity analysis are addressed (see paragraph 2)

# Section: Results

In the section head “Results”

1. **We have presented a clear description of participants in the study. As per the checklist we include:**
   1. The numbers of individuals at each stage of the study (see Figure 1)
   2. The reasons for non-participation (See Figure 1)
   3. We use a flow diagram to illustrate these points (See Figure 1)
2. **We provide descriptive data on participants, including:**
   1. Descriptions of characteristics of participants are presented in Table 1
   2. Participants with missing data, by variable, are identified in Table 1
3. **We provide outcome data over time, including:**

In the section head “Results” under the sub heading “The sample”

Numbers in each category are identified in paragraph 1, lines 1-3

In the section head “Results” under the sub headings “Cognitive outcomes” “Executive functions” and “Emotional and behavioural problems”

Paragraph 1, 2, 3 and Tables 2-5 outline for the reader the outcome data by measure

In the section head “Results” under the sub heading “Outcomes stratified by sex”

Paragraphs 1, 2, 3 and Tables 6-9 outline for the reader the outcome data stratified by sex

1. **We report the main results according to the checklist as follows:**

In the section head “Results” paragraphs 1-6 and in reference to Table 2-9:

- 1. We provide unadjusted estimates, confounder adjusted estimates and confidence intervals;
  2. We report category boundaries where cognition variables were categorised (see lines 255)
  3. Regarding population level risk analysis - in this case no population-level analysis is included

In section head “Methods” under sub heading “Statistical analysis” paragraph 1 lines 18-20 makes clear what confounders were identified from the literature and how they were included.

1. **We report other analyses conducted:**

In the section “Methods” under the subheading “Statistical analysis” in paragraph 2, and in reference to S1 Table, we provide additional analysis conducted.

# Section: Discussion

1. **We summarise results, and we reference to study objectives:**

In section head “Results” the last paragraph (paragraph 8) summarises results.

In the section head “Discussion”

Paragraph 2 reflects the main results linked to the study objectives

Paragraph 3 compares our results to those from other low-resource settings

1. **We present study limitations:**

Under the section head “Discussion” paragraph 8 outlines the limitation of the study.

1. **We give a cautious interpretation**

Under the section head “Discussion”

Paragraph 6 we offer a cautious interpretation of the findings in relation to similar findings and other relevant evidence

1. **We discuss the generalisability, link to previous research, and potential public health impact:**

In the section head “Discussion”

In paragraph 1 we outline the public health impact of conduct disorders

In paragraph 4 we outline the broader impact of executive function on children’s later health outcomes

In paragraph 3 and 9 we outline the strengths of the study and its unique contribution to the literature

# Section: Other information

In section head “Discussion” under the subheading “Acknowledgements” we identify the funder.
