# Supplementary material for: Exclusive Breastfeeding and Cognition, Executive Function, and Behavioural Disorders in Primary School-Aged Children in Rural South Africa: A Cohort Analysis
Source: PLoS Med. 2016 Jun 21;13(6):e1002044. doi: 10.1371/journal.pmed.1002044 (PMC4915617; doi:10.1371/journal.pmed.1002044)
Supplement: S1 Table — (DOCX) [file pmed.1002044.s002.docx]

**S1 Table:** Factors associated with children's cognitive and executive function *continuous* outcomes measured by the Kauffman Assessment Battery (KABC-II) and Developmental Neuropsychological Assessment (NEPSY)

|  | **Cognitive Assessment** | | | | | **Executive Function Assessment** | | |
| --- | --- | --- | --- | --- | --- | --- | --- | --- |
|  | **Sequential**  **(N=825)** | **Planning**  **(N=825)** | **Learning (N=825)** | **Simultaneous (N=825)** | **Riddles**  **(N=824)** | **Animal sorting (N=824)** | **Auditory attention (N=821)** | **Response set (N=820)** |
|  | **Beta [CI]** | **Beta [CI]** | **Beta [CI]** | **Beta [CI]** | **Beta [CI]** | **Beta [CI]** | **Beta [CI]** | **Beta [CI]** |
| ***Sex*** |  |  |  |  |  |  |  |  |
| Female | 0 | 0 | 0 | 0 | 0 | 0 | 0 | 0 |
| Male | -1.25*** [-1.9- -0.6] | -1.03*** [-1.5- -0.5] | 0.31 [-0.3-0.9] | 0.50 [-0.3-1.3] | -0.05 [-0.3-0.2] | -0.28 [-0.7-0.1] | -0.54* [-1.0- -0.1] | -0.30 [-0.7-0.1] |
| ***Child Age (Current)*** |  |  |  |  |  |  |  |  |
| 8 years | 0 | 0 | 0 | 0 | 0 | 0 | 0 | 0 |
| 9 years | -0.09 [-1.4-1.2] | 0.30 [-1.0-1.6] | 0.64 [-0.6-1.9] | -1.13 [-2.9-0.6] | -0.03 [-0.6-0.5] | 0.12 [-1.1-1.4] | 1.61*** [0.9-2.4] | 0.59 [-0.3-1.5] |
| 10 years | -0.26 [-1.6-1.1] | -0.08 [-1.4-1.3] | 0.48 [-0.8-1.8] | -1.70 [-3.5-0.1] | -0.38 [-0.9-0.2] | 0.38 [-0.9-1.7] | 2.23*** [1.5-3.0] | 0.50 [-0.4-1.4] |
| 11 years | -0.91 [-2.6-0.7] | 0.06 [-1.5-1.7] | 0.58 [-1.0-2.2] | -1.08 [-3.2-1.1] | -0.43 [-1.0-0.2] | 0.26 [-1.3-1.8] | 0.49 [-0.5-1.5] | -0.52 [-1.7-0.6] |
| ***Mother's Age (at birth)*** |  |  |  |  |  |  |  |  |
| Less than 20 years | 0 | 0 | 0 | 0 | 0 | 0 | 0 | 0 |
| 20-29 years | -0.21 [-1.2-0.8] | -0.41 [-1.2-0.4] | 0.15 [-0.7-1.0] | -0.19 [-1.3-0.9] | 0.13 [-0.2-0.4] | 0.63* [0.0-1.2] | 0.14 [-0.5-0.8] | -0.37 [-1.0-0.2] |
| 30+ years | 0.34 [-1.0-1.7] | -0.14 [-1.2-0.9] | 0.92 [-0.3-2.2] | -0.31 [-1.8-1.2] | 0.07 [-0.3-0.5] | 0.45 [-0.4-1.3] | 0.55 [-0.3-1.4] | -0.62 [-1.5-0.2] |
| ***Maternal IQ (Current)^a^*** |  |  |  |  |  |  |  |  |
| Low Raven’s | 0 | 0 | 0 | 0 | 0 | 0 | 0 | 0 |
| High Raven’s | 0.94** [0.3-1.6] | 1.05*** [0.5-1.6] | 0.91** [0.3-1.5] | 1.05* [0.2-1.9] | 0.42*** [0.2-0.6] | -0.14 [-0.6-0.3] | 0.28 [-0.2-0.8] | 0.05 [-0.4-0.5] |
| ***Mother's Education (at birth)*** |  |  |  |  |  |  |  |  |
| None | 0 | 0 | 0 | 0 | 0 | 0 | 0 | 0 |
| Primary | 0.56 [-0.9-2.0] | 0.04 [-1.2-1.3] | 0.36 [-0.7-1.5] | 0.53 [-1.2-2.3] | -0.11 [-0.5-0.3] | 0.12 [-0.8-1.0] | -0.75 [-1.8-0.3] | -0.01 [-0.9-0.9] |
| Some secondary | 0.97 [-0.6-2.5] | 0.55 [-0.7-1.8] | 0.70 [-0.5-1.9] | 0.76 [-1.1-2.6] | 0.15 [-0.3-0.6] | 0.56 [-0.4-1.5] | -0.16 [-1.3-1.0] | 0.70 [-0.3-1.7] |
| Completed secondary/post | 1.40 [-0.2-3.0] | 1.14 [-0.3-2.6] | 1.65* [0.3-3.0] | 1.65 [-0.4-3.7] | 0.21 [-0.3-0.7] | 0.75 [-0.3-1.8] | -0.07 [-1.2-1.1] | 0.76 [-0.3-1.8] |
| ***Birthweight*** |  |  |  |  |  |  |  |  |
| Low Birthweight | 0 | 0 | 0 | 0 | 0 | 0 | 0 | 0 |
| Normal Birthweight | 0.54 [-0.5-1.6] | 0.76 [-0.1-1.6] | 0.64 [-0.4-1.7] | 0.98 [-0.3-2.3] | 0.10 [-0.2-0.5] | 0.71 [-0.1-1.5] | 0.36 [-0.3-1.1] | 0.14 [-0.5-0.8] |
| ***Exclusive Breastfeeding*** |  |  |  |  |  |  |  |  |
| 0-1 months | 0 | 0 | 0 | 0 | 0 | 0 | 0 | 0 |
| 2-5 months | 0.69 [-0.4-1.7] | 0.46 [-0.4-1.3] | 0.47 [-0.5-1.4] | 0.68 [-0.5-1.9] | -0.03 [-0.4-0.3] | 0.30 [-0.5-1.1] | -0.29 [-1.1-0.5] | 0.04 [-0.7-0.8] |
| 6 months | 0.76 [-0.3-1.8] | 0.06 [-0.8-0.9] | 0.60 [-0.3-1.5] | 0.51 [-0.7-1.7] | 0.08 [-0.2-0.4] | 0.32 [-0.4-1.0] | -0.39 [-1.1-0.4] | -0.02 [-0.7-0.7] |
| ***Birth order (Birth)*** |  |  |  |  |  |  |  |  |
| Birth order 1-2 | 0 | 0 | 0 | 0 | 0 | 0 | 0 | 0 |
| Birth order 3-4 | -0.20 [-1.0-0.7] | -0.04 [-0.8-0.7] | -0.03 [-0.9-0.8] | 0.60 [-0.6-1.8] | 0.15 [-0.1-0.4] | -0.27 [-0.9-0.3] | -0.07 [-0.7-0.6] | 0.22 [-0.4-0.8] |
| Birth order 5+ | -0.27 [-1.5-0.9] | 0.05 [-1.0-1.1] | -0.54 [-1.8-0.7] | 1.13 [-0.4-2.7] | 0.20 [-0.2-0.6] | -0.17 [-1.1-0.7] | 0.14 [-0.7-1.0] | 0.41 [-0.4-1.3] |
| ***Mother's HIV status*** |  |  |  |  |  |  |  |  |
| Negative | 0 | 0 | 0 | 0 | 0 | 0 | 0 | 0 |
| Positive pregnancy | 0.40 [-0.4-1.2] | -0.25 [-0.9-0.4] | 0.24 [-0.4-0.9] | -0.32 [-1.2-0.6] | 0.07 [-0.2-0.3] | 0.07 [-0.4-0.6] | -0.17 [-0.7-0.4] | -0.06 [-0.6-0.5] |
| Positive since pregnancy | -0.00 [-0.9-0.9] | -0.77* [-1.4- -0.1] | 0.38 [-0.4-1.1] | -0.60 [-1.7-0.5] | -0.17 [-0.5-0.1] | -0.21 [-0.8-0.4] | -0.22 [-0.9-0.4] | 0.06 [-0.6-0.7] |
| ***Residence (at birth)*** |  |  |  |  |  |  |  |  |
| Rural | 0 | 0 | 0 | 0 | 0 | 0 | 0 | 0 |
| Urban | 0.40 [-0.3-1.1] | 0.17 [-0.4-0.8] | 0.62* [0.0-1.2] | 0.13 [-0.7-1.0] | 0.21 [-0.0-0.4] | 0.42 [-0.0-0.9] | -0.07 [-0.6-0.4] | 0.03 [-0.4-0.5] |
| ***Income provider (at birth)*** |  |  |  |  |  |  |  |  |
| Other | 0 | 0 | 0 | 0 | 0 | 0 | 0 | 0 |
| Mother | -0.10 [-1.2-1.0] | -0.01 [-0.8-0.8] | 0.18 [-0.8-1.2] | 0.52 [-0.8-1.8] | -0.06 [-0.4-0.3] | 0.43 [-0.4-1.2] | -0.33 [-1.2-0.6] | -0.04 [-0.9-0.8] |
| ***Owns fridge(at birth)*** |  |  |  |  |  |  |  |  |
| Fridge No | 0 | 0 | 0 | 0 | 0 | 0 | 0 | 0 |
| Fridge Yes | 0.37 [-0.3-1.0] | 0.68* [0.1-1.2] | 0.23 [-0.4-0.8] | 0.51 [-0.3-1.3] | 0.18 [-0.0-0.4] | 0.12 [-0.3-0.6] | -0.04 [-0.5-0.5] | 0.02 [-0.4-0.5] |
| ***Perception wealth(Current)*** |  |  |  |  |  |  |  |  |
| Very comfortable | 0 | 0 | 0 | 0 | 0 | 0 | 0 | 0 |
| Getting by | -1.39* [-2.5- -0.2] | 0.40 [-0.5-1.3] | -0.45 [-1.5-0.6] | 0.52 [-0.9-1.9] | -0.10 [-0.5-0.2] | 0.26 [-0.5-1.0] | 0.18 [-0.6-1.0] | -0.06 [-0.8-0.6] |
| Extremely poor | -1.24 [-2.5-0.0] | 0.12 [-0.8-1.0] | -0.55 [-1.6-0.5] | 0.38 [-1.1-1.9] | -0.19 [-0.6-0.2] | 0.19 [-0.6-1.0] | 0.55 [-0.3-1.4] | 0.42 [-0.4-1.2] |
| ***Creche*** |  |  |  |  |  |  |  |  |
| No creche | 0 | 0 | 0 | 0 | 0 | 0 | 0 | 0 |
| Attended creche | 1.04 [-0.0-2.1] | 0.21 [-0.8-1.2] | 0.07 [-1.0-1.2] | 0.13 [-1.4-1.7] | 0.06 [-0.3-0.4] | 0.47 [-0.3-1.3] | -0.33 [-1.2-0.6] | 0.18 [-0.6-1.0] |
| ***MC-Home^b^ (Current)*** |  |  |  |  |  |  |  |  |
| Low Total | 0 | 0 | 0 | 0 | 0 | 0 | 0 | 0 |
| High Total | 0.03 [-0.6-0.7] | 0.25 [-0.3-0.8] | 0.13 [-0.4-0.7] | 0.81* [0.0-1.6] | -0.04 [-0.3-0.2] | -0.07 [-0.5-0.4] | 0.53* [0.1-1.0] | 0.56* [0.1-1.0] |
| ***Maternal mental health (Current)*** |  |  |  |  |  |  |  |  |
| No mental disorders | 0 | 0 | 0 | 0 | 0 | 0 | 0 | 0 |
| Depression or anxiety or alcohol use | 0.52 [-0.7-1.7] | 0.13 [-0.7-1.0] | -0.55 [-1.6-0.5] | -0.30 [-1.7-1.1] | -0.15 [-0.5-0.2] | 0.27 [-0.5-1.0] | 0.67 [-0.2-1.5] | 0.21 [-0.5-0.9] |
| ***Parenting stress (Current)*** |  |  |  |  |  |  |  |  |
| Parenting stress ≤ 90 | 0 | 0 | 0 | 0 | 0 | 0 | 0 | 0 |
| Parenting stress ≥ 90 | -0.21 [-1.2-0.8] | -0.80* [-1.6- -0.1] | -0.34 [-1.2-0.5] | -0.87 [-2.0-0.3] | 0.01 [-0.3-0.3] | -0.37 [-1.1-0.3] | -0.44 [-1.1-0.3] | -0.39 [-1.0-0.2] |
| ***Intercept*** | 14.99*** [12.2-17.8] | 7.76*** [5.3-10.2] | 10.71*** [8.1-13.3] | 16.96*** [13.5-20.4] | 3.68*** [2.7-4.6] | 12.67*** [10.6-14.7] | 5.12*** [3.1-7.1] | 7.54*** [5.6-9.4] |

^a^ High/low based on splitting the sample on the median

^b^ High/low based on splitting the sample on the median

* p < 0.05; ** p < 0.01; *** p < 0.001.

Betas based on multivariate linear regression on the outcomes including all covariates

Sequential Processing: measures audio and visual memory and memory span

Simultaneous Processing: measures spatial and visual perception, reasoning and maths ability

Learning ability: measures focused and selective attention, and ability to store auditory and visual stimuli simultaneously

Planning: measures decision making ability

Riddles: measures reasoning and language development

Animal Sorting: measures inhibition, planning, cognitive flexibility

Auditory Attention: measures vigilance, selective/ sustained auditory attention

Response Set: measures inhibition of previously learned stimuli
